# Screening and identification of miR-181a-5p in oral squamous cell carcinoma and functional verification in vivo and in vitro

**DOI:** 10.1186/s12885-023-10600-3

**Published:** 2023-02-17

**Authors:** Guoqiang Xu, Yiyan Yang, Junting Yang, Lanfei Xiao, Xiaotang Wang, Litao Qin, Jiping Gao, Ruijing Xuan, Xiaofen Wu, Zhaoyang Chen, Rui Sun, Guohua Song

**Affiliations:** 1 Laboratory Animal Center, Shanxi Key Laboratory of Experimental Animal Science and Human Disease Animal Model, Shanxi Medical University, Road Xinjian 56, Taiyuan, 030001 China; 2 Shanxi Medical University School and Hospital of Stomatology, Taiyuan, 030001 China; 3 Shanxi Bethune Hospital, Shanxi Academy of Medical Sciences, Tongji Shanxi Hospital, Third Hospital of Shanxi Medical University, Taiyuan, 030032 China; 4 Shanxi Medical University School of Basic Medical Science, Taiyuan, 030001 China

**Keywords:** miRNAs, OSCC, Chinese hamster animal model, miR-181a-5p/BCL2 axis

## Abstract

**Background:**

Oral squamous cell carcinoma (OSCC) is a common malignant tumor associated with poor prognosis. MicroRNAs (miRNAs) play crucial regulatory roles in the cancer development. However, the role of miRNAs in OSCC development and progression is not well understood.

**Methods:**

We sought to establish a dynamic Chinese hamster OSCC animal model, construct miRNA differential expression profiles of its occurrence and development, predict its targets, and perform functional analysis and validation in vitro.

**Results:**

Using expression and functional analyses, the key candidate miRNA (miR-181a-5p) was selected for further functional research, and the expression of miR-181a-5p in OSCC tissues and cell lines was detected. Subsequently, transfection technology and a nude mouse tumorigenic model were used to explore potential molecular mechanisms. miR-181a-5p was significantly downregulated in human OSCC specimens and cell lines, and decreased miR-181a-5p expression was observed in multiple stages of the Chinese hamster OSCC animal model. Moreover, upregulated miR-181a-5p significantly inhibited OSCC cell proliferation, colony formation, invasion, and migration; blocked the cell cycle; and promoted apoptosis. *BCL2* was identified as a target of miR-181a-5p. *BCL2* may interact with apoptosis- (*BAX*), invasion- and migration- (*TIMP1*, *MMP2*, and *MMP9*), and cell cycle-related genes (*KI67*, *E2F1*, *CYCLIND1*, and *CDK6*) to further regulate biological behavior. Tumor xenograft analysis indicated that tumor growth was significantly inhibited in the high miR-181a-5p expression group.

**Conclusion:**

Our findings indicate that miR-181a-5p can be used as a potential biomarker and provide a novel animal model for mechanistic research on oral cancer.

**Supplementary Information:**

The online version contains supplementary material available at 10.1186/s12885-023-10600-3.

## Introduction

Oral squamous cell carcinoma (OSCC), the most common oral and maxillofacial malignancy, accounts for approximately 90% of oral cancers, with approximately 300,000 new cases reported each year worldwide [1]. OSCC usually originates from the bottom of the mouth, anterior two-thirds of the tongue, upper and lower alveoli, lips, and buccal mucosa [2]. Owing to its high tumor metastasis and recurrence, this life-threatening disease has a low 5-year survival rate [3]. Surgical treatment of OSCC may lead to functional disorders that seriously affect the overall appearance of a patient [4]. Moreover, OSCC is mainly diagnosed in the middle and late stages, making an in-depth analysis of the entire clinical OSCC process difficult. Owing to the specific location of OSCC and numerous side effects of surgical treatment, clinical samples are difficult to obtain. To discover new treatment options for OSCC, a better OSCC animal model must be established to improve our understanding of the molecular mechanisms of its formation and development.

The hamster buccal pouch (HBP) model is the most relevant animal model for studying human OSCC, including morphogenesis, phenotypic markers, and genetic alterations [5]. The HBP is covered by a thin layer of keratinized stratified squamous epithelium that is very similar in thickness to the mouth mucosa and the ventral surface of the tongue in humans [6]. The Chinese hamster (*Cricetulus griseus*) has a stronger vitality and smaller body (approximately 9 cm long) than other hamsters, enabling ease of operation with a single hand [7]. Altogether, Chinese hamsters have become ideal animals for research on oral diseases. Establishing an animal model of OSCC using Chinese hamsters would allow us to further research the pathogenesis of oral cancer.

Owing to their important regulatory roles in tumor development, non-coding RNAs have attracted extensive attention. MicroRNAs (miRNAs) are non-coding endogenous small RNAs, produced from the stem-loop regions of primary transcript miRNAs (pri-miRNAs) [8]. miRNAs can regulate cell apoptosis, proliferation, invasion, migration, and other biological behaviors and play a crucial role in several human cancers [9–11]. miRNAs are involved in various physiological and pathological processes in OSCC [12–14].

As a member of the miR-181a family, miR-181a-5p plays an important role in many diseases [15, 16]. miR-181a-5p acts as a tumor suppressor in gastric adenocarcinoma, papillary thyroid cancer, and hepatocellular carcinoma [13, 17, 18]. Goldi et al. reported that miR-181a represses NF-kB signaling and decreases cell proliferation and survival [19]. In aging-related studies, miR-181a-5p is significantly upregulated in senescent cells, indicating that high miR-181a-5p expression may induce cell aging, inhibit the cell cycle, and promote cell apoptosis [20, 21]. Liu et al. found that miR-181a reverses chemoresistance and inhibits Epithelial-mesenchymal transition (EMT) and metastatic potential in tongue squamous cell carcinoma cells [22]. Moreover, relevant literature reports that miR-181a is frequently downregulated in OSCC samples [23]. Thus, miR-181a-5p may act as an important tumor suppressor during tumorigenesis and development. Several studies show that miR-181a regulates key cell survival proteins and oncogenic signaling; therefore, we hypothesized that miR-181a could downregulate the expression of critical genes and signaling pathways that maintain the neoplastic state. Therefore, investigating the regulatory role of miR-181a-5p in the occurrence and development of OSCC is crucial. This study aimed to determine miR-181a-5p target genes and related pathways, construct potential miRNA-mRNA regulatory networks, and clarify new molecular mechanisms of OSCC.

We sought to establish a dynamic Chinese hamster OSCC animal model with simple hyperplasia, abnormal hyperplasia, and squamous cell carcinoma groups; construct miRNA expression profiles; and employ high-throughput sequencing to further study the mechanisms underlying OSCC development by miRNAs. We collected OSCC specimens and multiple human OSCC cell lines to verify the expression of miR-181a-5p. To further identify the role of key differential miRNAs (miR-181a-5p) in the animal model, we performed a biological function experiment with a human OSCC cell line. Additionally, a tumor xenograft animal model was established to evaluate the effects of miR-181a-5p on tumor growth.

From the perspective of comparative medicine, the findings herein could provide new approaches and scientific evidence to support the notion that miRNA mutations lead to disorders in the molecular mechanisms that give rise to oral cancer.

## Materials and methods

### Research of OSCC animal model and screening of candidate miRNAs

#### Establishment of the animal model

Ninety male Chinese hamsters (*C. griseus*, 8–10-week-old, 21–25 g body weight) were provided by the Experimental Animal Center of Shanxi Medical University (Taiyuan, China SCXK Jin 2019-0004), housed in standard hamster cages in a barrier environment (25 °C, 45% humidity, 12/12 h light/dark cycles), and fed standard hamster diet and water ad libitum (SYXK Jin 2019-0007).

The protocol was approved by the Institutional Animal Care and Use Committee of Shanxi Medical University (IACUC 2017-018). Animals were randomly divided into control (n = 30), solvent control (n = 15), and treatment groups (n = 45). The control group was not subjected to treatment, while the treatment and solvent control groups were coated with a bilateral buccal pouch with 0.5% DMBA (7, 12-dimethylbenz(a)anthracene; Sigma, USA) or acetone (Sigma, USA) solution three times per week. The doses were selected based on previous studies [4, 24, 25]. To observe carcinogenesis, we collected samples at 6, 9, 12, 15, and 18 weeks for histopathological examination. The pathological changes in the buccal pouch were determined according to the 12-grade record reported by the World Health Organization (WHO) standard [26].

#### Histopathological examination

Buccal pouch samples were fixed in 4% paraformaldehyde (Boster, China) for approximately 48 h, dehydrated in gradient alcohol, diaphanized in xylene, embedded in paraffin, sectioned into 4 μm thick sections, and stained with hematoxylin and eosin (HE). Histopathological specimens were examined under a light microscope by an oral pathologist.

#### Ultrastructure examination

After sectioning into 1 mm^3^ pieces, the samples were fixed in 2.5% glutaraldehyde at 4 °C. The tissues were treated with a series of concentrations of ethanol, embedded in epoxy resin, trimmed, and sectioned. After staining with uranyl acetate and lead citrate, the samples were examined and photographed using a JEM-1011 transmission electron microscope (Tokyo, Japan).

#### RNA sample preparation and quality control

The samples for sequencing and qRT-PCR were immediately isolated, frozen in liquid nitrogen, and stored at -80 °C. The samples were divided into four grades according to pathological and ultrastructural examination results: control (normal), simple hyperplasia, abnormal hyperplasia, and squamous cell carcinoma. Samples from each group were randomly selected for the construction of RNA libraries for sequencing. Total RNA was extracted using the TRIzol reagent (Invitrogen, USA). The purity and integrity were monitored using a NanoPhotometer® spectrophotometer (Implen, GER) and an Agilent2100 Bioanalyzer (Agilent, USA).

#### RNA library construction and deep sequencing

After the RNA quality test, 18–30 nt RNA fragments were selected, purified, and ligated with 5′- and 3′-adaptors. Reverse transcription was used to create cDNA, and the amplified cDNA was purified from an agarose gel and amplified by PCR. Finally, the samples were sequenced using an Illumina HiSeq2500 Analyzer (Illumina, CA, USA).

#### Differential expression analysis

DEseq2 was used to analyze the differentially expressed miRNAs (DEmiRNAs). We corrected for multiple comparisons and used the adjusted P value < 0.05 and log_2_|(foldchange)| > 1 as the threshold.

#### Functional analysis of differentially expressed genes

ClusterProfiler was used to perform enrichment analysis of Gene Ontology (GO) annotation and Kyoto Encyclopedia of Genes and Genomes (KEGG) pathways [27]. A corrected P value < 0.05 was used as the threshold.

#### Quantitative real-time PCR

Total RNA was isolated from the tissues and cell lines using TRIzol reagent (Takara, Japan). miRNA cDNA was generated using the Mir-X™ miRNA First Strand Synthesis Kit (Takara, Japan), and the cDNA from mRNA was synthesized using the Prime Script™ RT Master Mix kit (Takara, Japan), according to the manufacturer’s protocol.

qRT-PCR was performed using the SYBR Green PCR Master Mix kit (Takara, Japan) on a StepOnePlus system (ABI, USA). The primers synthesized by Takara are listed in Supplementary Table 1. U6 and GAPDH were used as references.

#### Target gene prediction and gene expression profiling interactive analysis (GEPIA)

miRanda (v3.3a) [28] was used to predict target genes. miRTarBase [29] was used to predict the target genes of miR-181a-5p for further verification. The GEPIA database (http://gepia.cancer-pku.cn/index.html) was used to generate survival curves and perform correlation analyses.

### Expression analysis of candidate miRNA in samples from patients with OSCC and cell lines and in vitro functional validation

#### Patient sample collection

Five OSCC samples and three normal counterparts with pathologically definitive diagnoses were collected from Shanxi Provincial People’s Hospital. All samples were from males, and one case had lymph node metastasis. The specific clinical features are shown in Supplementary Table 2. The patients provided written informed consent for the use of their tissues for research purposes. The experimental research was approved by the Ethics Committee of Shanxi Provincial People’s Hospital (2019-05).

#### Cell culture

The human OSCC cell lines CAL-27 and SCC-25 were purchased from Procell Life Science and Technology Co., Ltd. (Hubei, China). The HOK cell line was provided by the School and Hospital of Stomatology at Shanxi Medical University. The CAL-27 cell line was grown in Dulbecco’s Modified Eagle Medium (DMEM, Boster, China), the SCC-25 cell line was grown in DMEM/Nutrient Mixture F-12 (DMEM/F12, Boster, China), and the HOKs were grown in Hyclone1640 medium (Hyclone, USA). The medium contained 10% fetal bovine serum (Gibco, USA) and 1% penicillin-streptomycin solution (Solarbio, China) and was maintained in an incubator with 5% CO_2_ at 37 °C.

#### Cell transfection

The miR-181a-5p mimics (5’-AACAUUCAACGCUGUCGGUGAGU-3’ and 5’-UCACCGACAGCGUUGAAUGUUUU-3’), miR-181a-5p inhibitor (5’-ACUCACCGACAGCGUUGAAUGUU-3’), and their corresponding negative controls (NCs) (miR-181a-5p mimics NC: 5’-UUCUCCGAACGUGUCACGUTT-3’ and 5’-ACGUGACACGUUCGGAGAATT-3’ and miR-181a-5p inhibitor NC: 5’-CAGUACUUUUGUGUAGUACAA-3’) were purchased from GenePharma Inc. (Shanghai, China). The cell line was transfected with the above sequence for 48 h using Lipofectamine™ RNAiMAX (Invitrogen). A transfection efficiency assay was performed after 48 h using qRT-PCR.

#### Cell proliferation

Cells were seeded in 96-well plates at 3 × 10^3^ cells/well and cultured for 0, 24, 48, and 72 h. Cell Counting Kit-8 (Boster, China) was used to detect cell proliferation according to the manufacturer’s protocol.

#### Colony formation

At 48 h after transfection, the transfected cells were harvested, and 2000 cells were seeded into each well of a 6-well plate. The cells were then cultured for 15 days in an incubator at 37 °C and 5% CO_2_. Thereafter, the cells were washed twice with phosphate-buffered saline (PBS), fixed with 4% paraformaldehyde for 30 min, and stained with 0.1% crystal violet for 30 min. Surviving colonies (≥ 50 cells) were counted.

#### Cell apoptosis

The cells (approximately 1 × 10^6^) were transfected for 48 h, harvested with trypsin, washed, and suspended in PBS. Subsequently, the cells were stained using the Annexin V-PE/7-AAD apoptosis detection kit (KeyGen Biotech, Nanjing, China). A FACS Arial II flow cytometer (BD Biosciences) was used to determine the rate of apoptosis.

#### Cell cycle

After transfection for 48 h, the cells were harvested and fixed in 70% ice-cold ethanol at 4 °C overnight. The cells were resuspended in PBS and stained using a cell cycle detection kit (KeyGen Biotech, Nanjing, China). A FACS Arial II flow cytometer (BD Biosciences) was used to detect the cell cycle.

#### Cell invasion

Cell invasion assays were performed using a transwell chamber (Corning, USA). Briefly, the transwell chamber was coated with 100 µL Matrigel™ (Corning, USA) and dried for approximately 1 h in an incubator. Cells (2 × 10^4^) were transfected for 48 h, resuspended in 150 µL serum-free medium, and seeded into the upper chambers; 650 µL of medium with 10% serum was added to the lower chamber. The cells were then incubated for 24 h in an incubator, and the upper sides of the chamber were gently scraped with a cotton swab to remove non-invasive cells. The invading cells on the lower sides were fixed in 4% paraformaldehyde, stained with 0.1% crystal violet, photographed, and counted under a light microscope (×200).

#### Cell migration

Transwell chambers (Corning, USA) were used to assess cell migration. For cell migration, the transwell chamber was not coated with Matrigel™, and the remaining steps were the same as those used for cell invasion.

We also detected cell migration using a scratch test. OSCC cells were inoculated into a 6-well plate. When the cell fusion degree reached 80–90%, a sterile 200-µL pipette tip was used to scratch. The old medium was pipetted, and PBS was used to wash the floating cells and residual medium. Fresh medium containing 1% serum was then added, and the cells were cultivated in an incubator. The area between the wounds was measured at different time points, and the relative migration rate was calculated.

#### Western blotting

Total protein was extracted using a protein extraction kit (Boster, China). The concentration was determined using a BCA kit (Boster, China). SDS-PAGE was used to separate the protein samples. The proteins were transferred onto a polyvinylidene difluoride (PVDF) membrane. After incubation on a shaker with blocking solution, the PVDF membrane was incubated overnight at 4 °C with the following primary antibodies: TIMP1 (1:600 dilution; Abcam, USA), BCL2 (1:800 dilution; Proteintech, China), and β-actin (1:1000 dilution; Cell Signaling Technology, USA). The membrane was subsequently washed and incubated with the target secondary antibody (1:5000 dilution; Boster, China) for 1 h at 37 °C. Finally, the bands were measured and photographed using an enhanced chemiluminescence system (Box Chemi XX9, UK).

#### Construction of the PPI network of *TIMP1*

To further study the interaction of *TIMP1*, the STRING database (http://string.embl.de/) was used to generate protein–protein interaction (PPI) networks.

### In vivo functional validation of candidate miRNAs by xenograft models

#### Construction of the stable cell line

CAL-27 cells grown to 30–50% confluence were transfected with either miR-181a-5p (OE) or Con (negative control, NC) lentiviral vector labeled with luciferase. After 48 h of recovery, the cells were subjected to puromycin selection to obtain two stable CAL-27 cell lines, overexpressing miR-181a-5p (OE) and OE-Con (negative control, NC).

#### Tumor xenograft animal model and imaging

Twenty-four-week-old male BALB/c nude mice (18–22 g) were purchased from the Beijing Vital River Laboratory Animal Technology Co., Ltd (Beijing, China) and housed in the Experimental Animal Center of Shanxi Medical University (Taiyuan, China SCXK Jin 2019-0007). The study protocol was approved by the Institutional Animal Care and Use Committee of Shanxi Medical University (IACUC SYDL2021013). Twenty nude mice were randomly divided into the NC and OE groups (n = 10 per group). The stable cell lines NC and OE (5 × 10^6^) were subcutaneously inoculated into the left armpit of mice. The activity, diet, and mental state of the animals were observed daily. The tumor volume and weight of the mice were measured using a caliper and electronic scale every two days and calculated using the formula length × width^2^ × 1/2. Finally, the mice were euthanized (cervical dislocation) 40 days after the injection, and the tumors were photographed and dissociated.

To better evaluate tumor growth in nude mice, luciferase activity in tumor tissue was detected using the IVIS LuminaXR system (Perkin Elmer, Norwalk, Connecticut, USA).

### Statistical analysis

All data were analyzed using the IBM SPSS Statistics 24 software. The LSD t-test was used to compare groups. All values are expressed as mean ± standard error of the mean, and P < 0.05 was used to indicate statistical significance.

## Results

### Establishment of OSCC chinese hamster animal model and identification and screening of miR-181a-5p

#### Histopathological changes in oral buccal pouch mucosa

**Fig. 1:**
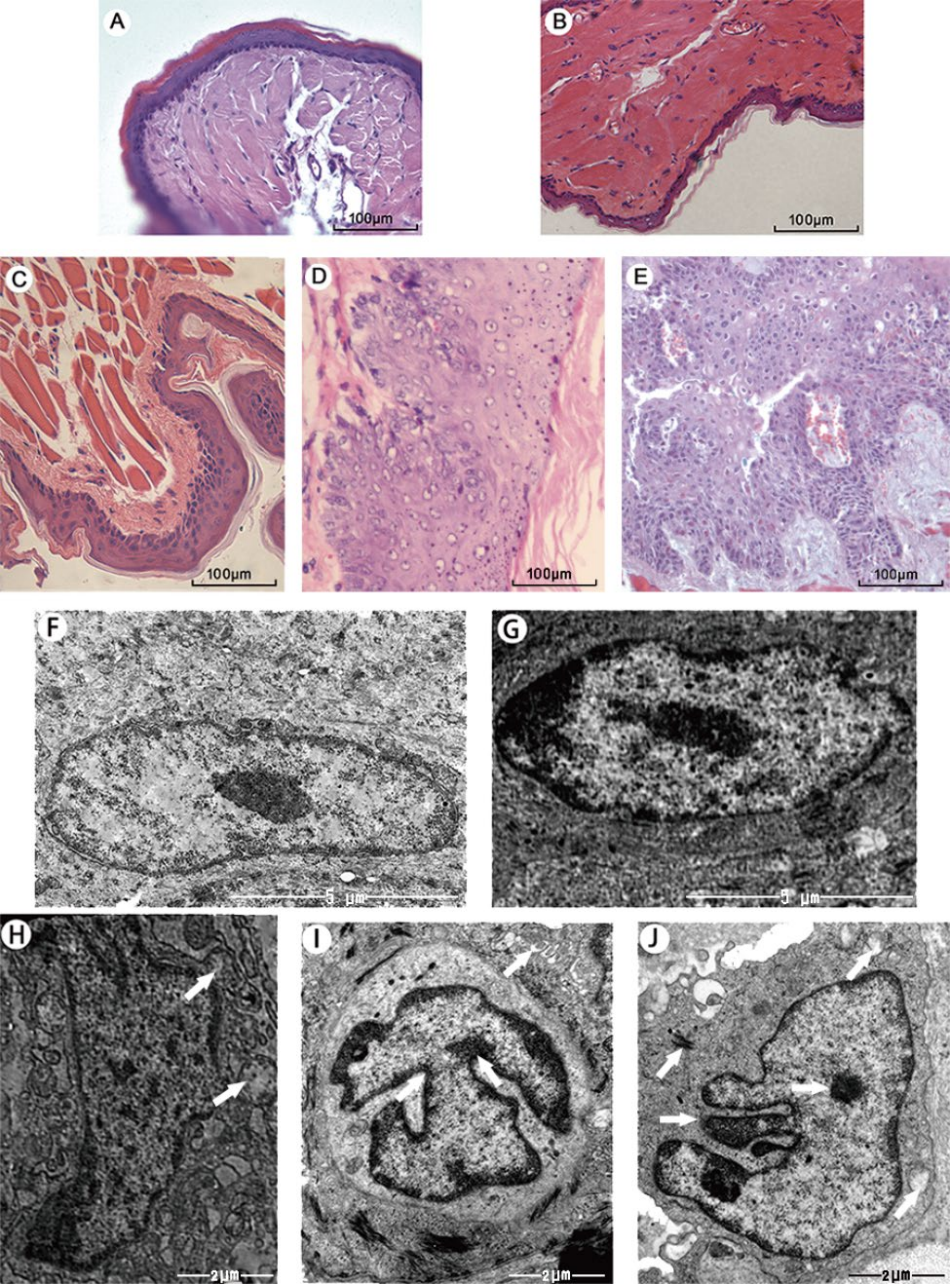
**Pathological (A-E) and ultrastructure (F-J) analysis. (A)** Control group; **(B)** Solvent control group; **(C)** Simple hyperplasia group; **(D)** Abnormal hyperplasia group; and **(E)** Squamous cell carcinoma group. Scale = 100 μm. **(F)** Control group; **(G)** Solvent control group; (Scale = 5 μm) **(H)** Simple hyperplasia group; **(I)** Abnormal hyperplasia group; and **(J)** Squamous cell carcinoma group

Based on the 12 grades listed by the WHO for cancer diagnosis, we divided the animal model into four stages: normal, simple hyperplasia, abnormal hyperplasia, and squamous cell carcinoma. No significant differences were observed between the solvent control and control groups (Fig. 1A, B). However, in the simple hyperplasia stage, the granular and spinous layers of the mucous epithelium became thick and displayed the shape of a papillate or nail, with inflammatory cells infiltrating the mucous membrane (Fig. 1C). In the abnormal hyperplasia stage, the nuclei were irregular, abnormally proliferating cells in the epithelial layer were increased, and the granular and spinous layers of the mucous epithelium were further thickened (Fig. 1D). In the squamous cell carcinoma stage, the cells permeated through the basement membrane, infiltrating the lamina propria and connective tissue. Many tumor islands emerged at this stage. The tumor cells showed atypical mitosis accompanied by keratinization and nuclear enlargement (Fig. 1E).

#### Changes in the ultrastructure of oral buccal pouch mucosa

Similar to the histopathological diagnosis, no significant ultrastructural changes were observed in the solvent control and control groups (Fig. 1F, G). In the simple hyperplasia stage, the intercellular space was slightly increased, the morphologies of the cell and nucleus were regular, mitochondria increased, partial enlargement was observed, and desmosomes were reduced (Fig. 1H). In the abnormal hyperplasia stage, the cells were enlarged, intercellular spaces were further widened, the morphology of the nucleus was irregular and enlarged, nucleoli gathered at the edge, nuclear-to-plasma ratio increased, desmosomes were reduced, mitochondria appeared edematous, and some mitochondrial ridges disappeared (Fig. 1I). When the squamous cell carcinoma stage was reached, the cell volume increased, the shape of the cells was irregular, the nucleus condensed into irregular jagged structures, nucleoli were concentrated, desmosomes were further reduced or even disappeared, and mitochondria were swollen with vacuoles (Fig. 1J).

#### Differential expression analysis of the miRNAs

**Fig. 2:**
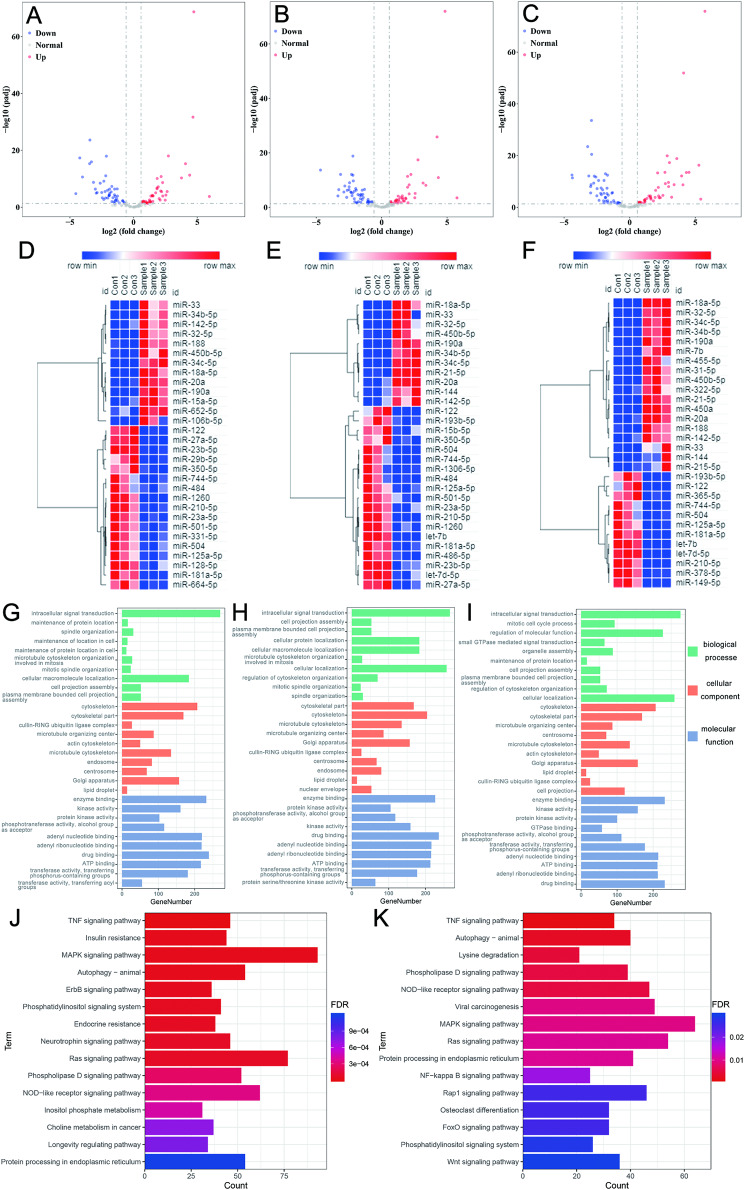
(**A-C**) Volcano plot of dysregulated miRNAs: **(A)** Simple hyperplasia group; **(B)** Abnormal hyperplasia group, and **(C)** Squamous cell carcinoma group; Vertical axis correspond to –log(padj). Horizontal axis represents log_2_ (fold change). The red and blue dots represent upregulated and downregulated miRNAs, respectively. **(D-F)** Heatmap of differentially expressed miRNAs: **(D)** Simple hyperplasia group; **(E)** Abnormal hyperplasia group; and **(F)** Squamous cell carcinoma group. **(G-I)** GO classification of the target genes: **(G)** Simple hyperplasia group; **(H)** Abnormal hyperplasia group; and **(I)** Squamous cell carcinoma group. **(J-K)** KEGG analysis of the target genes: **(J)** Top 15 significantly enriched pathways between the simple hyperplasia and Squamous cell carcinoma groups; **(K)** Top 15 significantly enriched pathways between the simple hyperplasia stage and Squamous cell carcinoma groups. The colors indicate the FDR value of the pathways

Seventy-seven DEmiRNAs were identified in the simple hyperplasia group. Of these, 42 (54.55%) were downregulated, and 35 (45.45%) were upregulated (Fig. 2A). Furthermore, 76 DEmiRNAs were identified in the abnormal hyperplasia group. Of these, 43 (56.58%) were downregulated, and 33 (43.42%) were upregulated (Fig. 2B). Finally, 76 DEmiRNAs were identified in the squamous cell carcinoma group. Of these, 39 (51.32%) were downregulated, and 37 (48.68%) were upregulated (Fig. 2C). Sixty miRNAs were also recognized to be differentially expressed in all three stages, indicating that these miRNAs may play a crucial role in the entire process of occurrence and development of oral cancer and could thus be the focus of future functional research. The top 30 significantly altered genes in the three groups are separately shown in Fig. 2D-F, and information on the top 30 altered genes in all three groups is provided in Supplementary Table 3.

At the same time, we also compared the simple hyperplasia, abnormal hyperplasia, and squamous cell carcinoma groups to identify their DEmiRNAs. Only one DEmiRNA was upregulated between the abnormal hyperplasia and simple hyperplasia groups (Supplementary Table 4). We screened 35 DEmiRNAs between the squamous cell carcinoma and simple hyperplasia groups; of these, 21 were upregulated, and 14 were downregulated (Supplementary Table 5). Moreover, we screened 18 DEmiRNAs between the squamous cell carcinoma and abnormal hyperplasia groups; of these, 11 were upregulated, and 7 were downregulated (Supplementary Table 6).

#### GO enrichment

The targets of DEmiRNAs in the three groups were classified according to GO classification (Fig. 2G-I). Accordingly, 11 biological processes, 24 cell components, and 42 molecular functions were projected into the simple hyperplasia group; 10 biological processes, 36 cell components, and 43 molecular functions were projected into the abnormal hyperplasia group; and 42 biological processes, 18 cell components, and 48 molecular functions were projected into the squamous cell carcinoma group.

Further analysis revealed that there were nine (19%) entries in all three stages of the biological process, and these stages were predominantly involved in intracellular signal transduction, cellular macromolecule localization, and cellular protein localization. In the cellular component, there were 11 (34.4%) entries in the three stages, and the target genes in the three stages were largely responsible for the cytoskeleton, cytoskeletal parts, and the Golgi apparatus. In terms of molecular function, there were 37 entries (69.8%). Furthermore, the target genes in the three stages were mainly enriched for enzyme binding, adenyl nucleotide binding, adenyl ribonucleotide binding, drug binding, and ATP binding.

#### KEGG enrichment

We found 107, 107, and 110 significantly enriched pathways in simple hyperplasia, abnormal hyperplasia, and squamous cell carcinoma stages, respectively. Further, the targets of DEmiRNAs in the three groups were mainly enriched in the TNF signaling pathway, autophagy–animal, axon guidance, the MAPK signaling pathway, the Ras signaling pathway, and the AMPK signaling pathway. Ninety-eight signaling pathways were significantly enriched in all three stages. Information on the top 15 enriched pathways in the three stages is provided in Supplementary Table 7.

To further elucidate the genes and pathways that may influence the occurrence and development of OSCC, we analyzed the different signaling pathways between the precancerous and cancer stages. In the simple hyperplasia stage group, the targets of DEmiRNAs were mainly enriched in the TNF signaling pathway, autophagy, and the MAPK signaling pathway compared to those in the squamous cell carcinoma stage group. Further, in the abnormal hyperplasia stage group, the targets of DEmiRNAs were mainly enriched in the TNF, autophagy, and lysine degradation signaling pathway compared to those in the squamous cell carcinoma stage group. The top 15 significantly enriched pathways in the two groups are shown in Fig. 2J K.

#### Verification of differentially expressed miRNAs by qRT-PCR

**Fig. 3:**
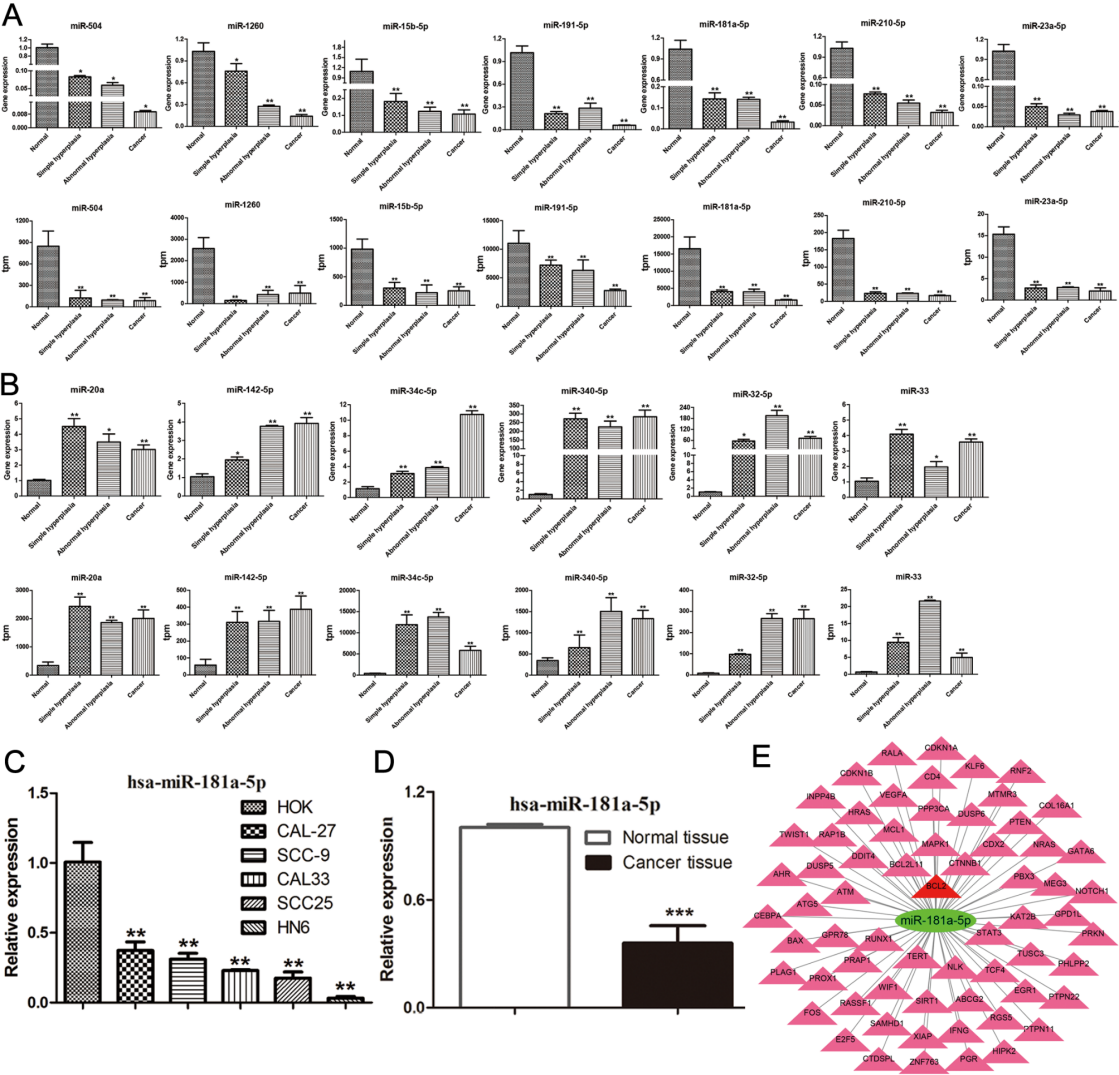
**(A)** Low-expressed miRNAs; the first line represents miRNA expression level in the four groups detected using qRT-PCR (n = 5), and the second line represents miRNA expression level detected using transcriptome sequencing (n = 3); **(B)** High-expressed miRNAs; the third line represents miRNA expression level in the four groups detected using qRT-PCR (n = 5), and the fourth line represents miRNA expression level detected using transcriptome sequencing (n = 3); **(C)** The expression of miR-181a-5p in HOK and OSCC cell lines (n = 3); **(D)** The expression of miR-181a-5p in human normal and OSCC tissues; **(E)** The interaction between miR-181a-5p and its main target genes

Compared to those in the normal group, the expression levels of miR-504-5p, miR-1260, miR-15b-5p, miR-191-5p, miR-181a-5p, miR-210-5p, and miR-23a-5p decreased in all three stages (Fig. 3A). The expression of miR-20a, miR-142-5p, miR-34c-5p, miR-340-5p, miR-32-5p, and miR-33 in the three stages was significantly upregulated (Fig. 3B) compared to that in the normal group. In general, miRNA expression detected by qRT-PCR and RNA sequencing was highly consistent, indicating the reliability of the sequencing data.

#### Identification of the miRNA for subsequent studies

Combining the sequencing and qPCR verification results, we found that miR-181a-5p gradually downregulated in OSCC and reached a minimum level at the squamous cell carcinoma stage. After searching miRbase, we found that miR-181a-5p is highly conserved in mammals, which is consistent with previous findings [23]. Moreover, its mature sequences were identical between humans and our animal model. However, little is known regarding the role of miR-181a-5p in oral cancer. Therefore, miR-181a-5p was selected as a candidate gene for the subsequent in vitro validation.

### miR-181a-5p expression is downregulated in specimens of patients with OSCC and cell lines

The human cell line detection results indicated that the expression of miR-181a-5p in human OSCC cell lines was significantly lower than that in the HOK cell line (Fig. 3C). Furthermore, the human specimen validation results showed that the expression of miR-181a-5p in OSCC tissue was significantly lower than that in the normal tissue (Fig. 3D). The interaction between miR-181a-5p and its main targets, validated in previous studies, is shown in Fig. 3E.

### miR-181a-5p affects the biological behavior of OSCC cells in vitro

#### Transfection efficiency detection

**Fig. 4:**
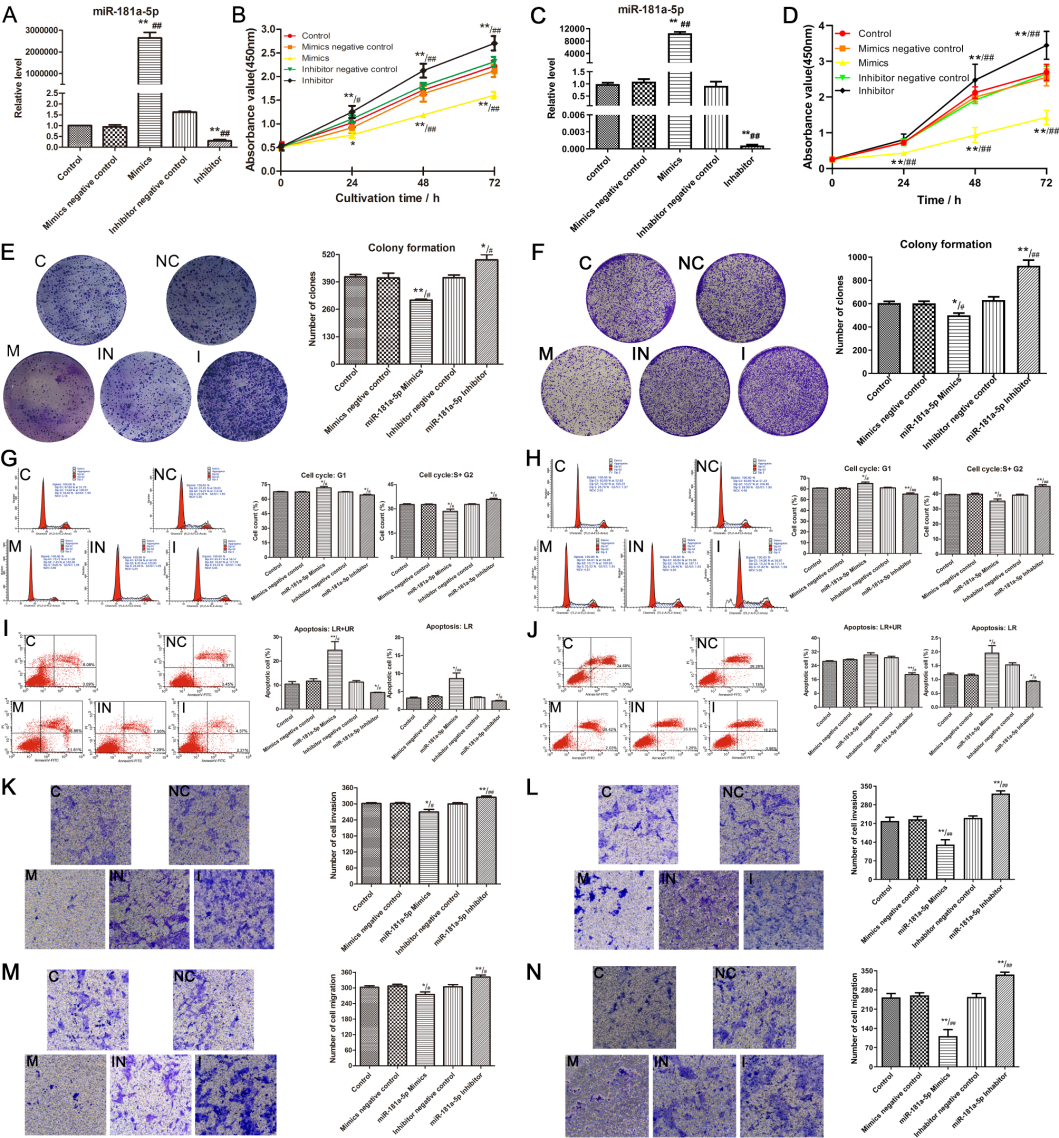
**(A)** Verification of transfection efficiency in the CAL27 cell line; **(B)** Detection of cell proliferation in the CAL27 cell line; **(C)** Verification of transfection efficiency in the SCC25 cell line; **(D)** Detection of cell proliferation in the SCC25 cell line; **(E)** Detection of cellular colony formation in the CAL27 cell line; **(F)** Detection of cellular colony formation in the SCC25 cell line; **(G)** Detection of cell cycle in the CAL27 cell line (n = 3); **(H)** Detection of cell cycle in the SCC25 cell line; **(I)** Detection of apoptosis in the CAL27 cell line (n = 3); **(J)** Detection of apoptosis in the SCC25 cell line (n = 3); **(K)** Detection of invasion in the CAL27 cell line (n = 3); **(L)** Detection of invasion in the SCC25 cell line (n = 3); **(M)** Detection of migration in the CAL27 cell line (n = 3); **(N)** Detection of migration in the SCC25 cell line (n = 3). C, blank control group; NC, mimics negative control group; M, mimics group; IN, inhibitor negative control group; I, inhibitor group. * P < 0.05 and ** P < 0.01 vs. Control group; # P < 0.05 and ## P < 0.01 vs. mimics / inhibitor negative control group

To investigate the mechanism of miR‑181a-5p in OSCC, CAL-27 and SCC-25 cell lines were transfected with the corresponding sequences. In the mimic group, the expression of miR-181a-5p was significantly upregulated compared to that in the control and NC group; however, in inhibitor group, miR-181a-5p expression was significantly downregulated compared to that in the control and NC group. No significant differences were found between the control and NC groups (Fig. 4A, C).

#### Altered miR-181a-5p expression modulates the proliferation and colony formation of the OSCC cell line

Based on the CCK-8 assay, miR-181a-5p overexpression significantly inhibited proliferation in the mimic group compared to that in the NC and control groups, whereas downregulation of miR-181a-5p promoted proliferation in the two cell lines (Fig. 4B, D). Furthermore, the colony formation assay also indicated that miR-181a-5p overexpression significantly inhibited colony formation, whereas downregulation of miR-181a-5p promoted colony formation in the inhibitor group compared to that in the NC and control groups (Fig. 4E, F). These results were consistent between the two OSCC cell lines.

#### Altered miR-181a-5p expression regulates the cell cycle and apoptosis of the OSCC cell line

OSCC cell lines treated with miR-181a-5p mimics were arrested in the G1 phase, and the cell population in the G2 and S phases was reduced relative to those treated with NC and control. In contrast, transfection with the miR-181a-5p inhibitor reduced the cell population in the G1 phase and increased the cell population in the G2 and S phases (Fig. 4G, H).

Apoptosis was measured using flow cytometry. Accordingly, the apoptotic rate increased when the cells were transfected with miR-181a-5p mimics; however, the miR-181a-5p inhibitor decreased the apoptotic rate (Fig. 4I, J).

#### miR-181a-5p inhibits OSCC cell invasion and migration

Transwell chamber assay revealed that miR-181a-5p mimics significantly reduced cell invasion compared to NC and control; however, the miR-181a-5p inhibitor markedly increased cell invasion in the two OSCC cell lines (Fig. 4K, L). The migration assay also demonstrated that overexpression of miR-181a-5p inhibited the migration of OSCC cells, whereas downregulation of miR-181a-5p promoted cell migration (Fig. 4M, N).

**Fig. 5:**
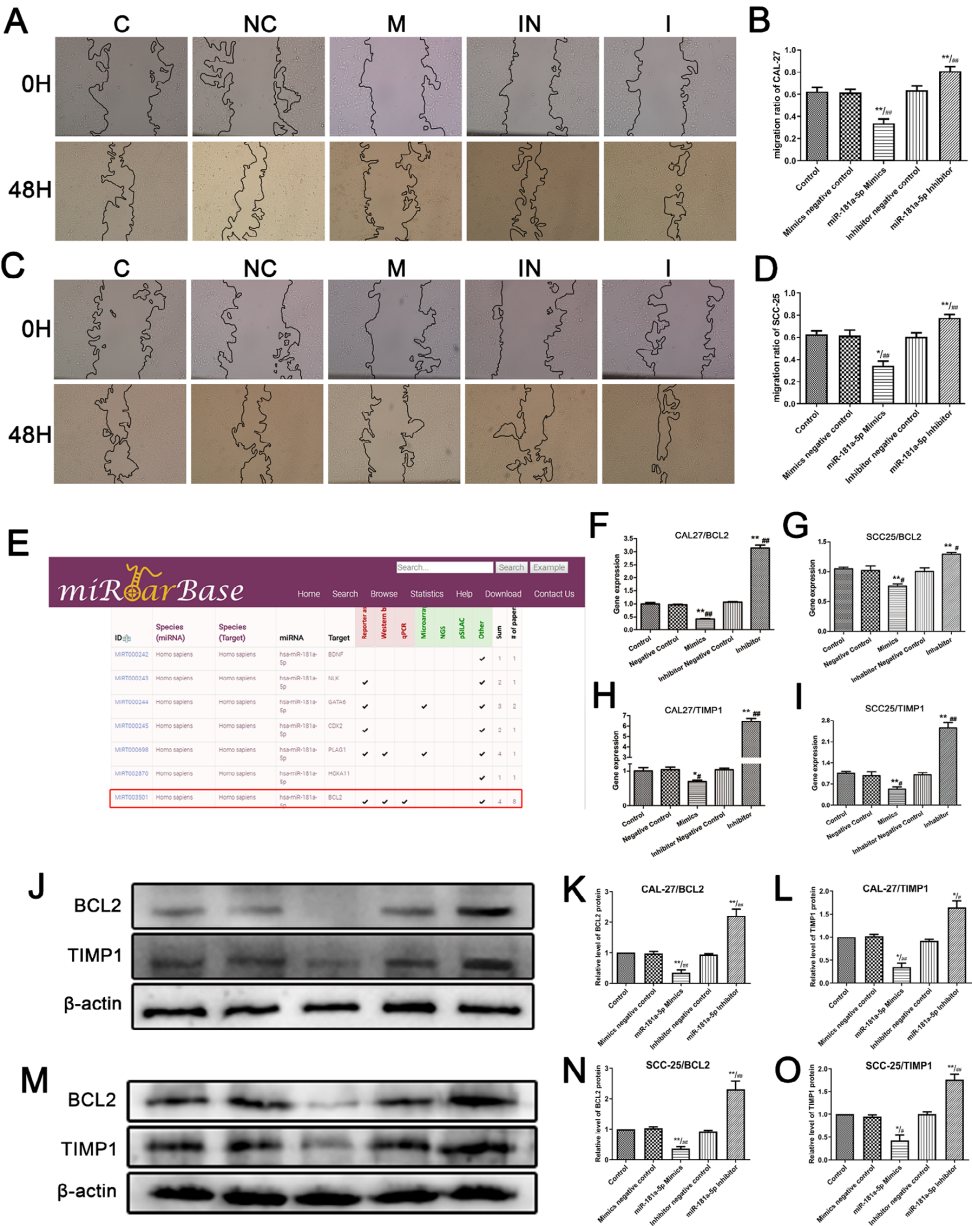
**(A)** Scratch assays of CAL27 cell line; **(B)** Statistical results of scratch assays of CAL27 cell line; **(C)** Scratch assays of SCC25 cell line; **(D)** Statistical results of scratch assays of SCC25 cell line; **(E)** Target gene prediction in miRTarbase; **(F, G)** The mRNA expression of target gene *BCL2* in CAL27/ SCC25 cell line (n = 3); **(H, I)** The mRNA expression of *TIMP1* in CAL27/SCC25 cell line (n = 3); **(J)** The protein expression in CAL27 cell line; **(K)** Relative protein level of BCL2 in CAL27 cell line; **(L)** Relative protein level of TIMP1 in CAL27 cell line; **(M)** The protein expression in SCC25 cell line; **(N)** Relative protein level of BCL2 in SCC25 cell line; **(O)** Relative protein level of TIMP1 in SCC25 cell line; * P < 0.05 and ** P < 0.01 vs. Control group; ^#^ P < 0.05 and ^##^ P < 0.01 vs. the mimics/inhibitor negative control group. We cropped the blots before antibody hybridization. The full-length membranes, with membrane edges visible, are presented in Supplementary Fig. 1

The scratch assay depicted that the miR-181a-5p inhibitor could promote the migration of CAL-27 and SCC-25 cells (Fig. 5A-D). However, the wound healing rate was lower in the miR-181a-5p mimics group than that in the NC and control groups, indicating that miR-181a-5p could inhibit cell migration. Moreover, the two OSCC cell lines showed consistent results. The results revealed that miR-181a-5p overexpression and knockdown had an inhibitory and facilitative effect, respectively, on OSCC cell migration and invasion.

#### Screening and confirming the target genes of miR-181a-5p

We used miRTarBase to screen for the target genes of miR-181a-5p. miRTarBase is a well-known public database that contains experimentally verified miRNA target genes. According to miRTarBase, *BCL2* is the target gene of miR-181a-5p; this was verified using reporter assay, western blotting, and qPCR (Fig. 5E). We also verified the inhibitory effects of miR-181a-5p (Fig. 5F, G, J, K, M, and N). Based on our previous findings, overexpression of miR-181a-5p inhibited *BCL2* mRNA and protein levels. Moreover, miR-181a-5p also affected the mRNA and protein expression of *TIMP1* (Fig. 5H, I, J, L, M, and O).

#### Interaction and prognosis analysis of the downstream genes

**Fig. 6:**
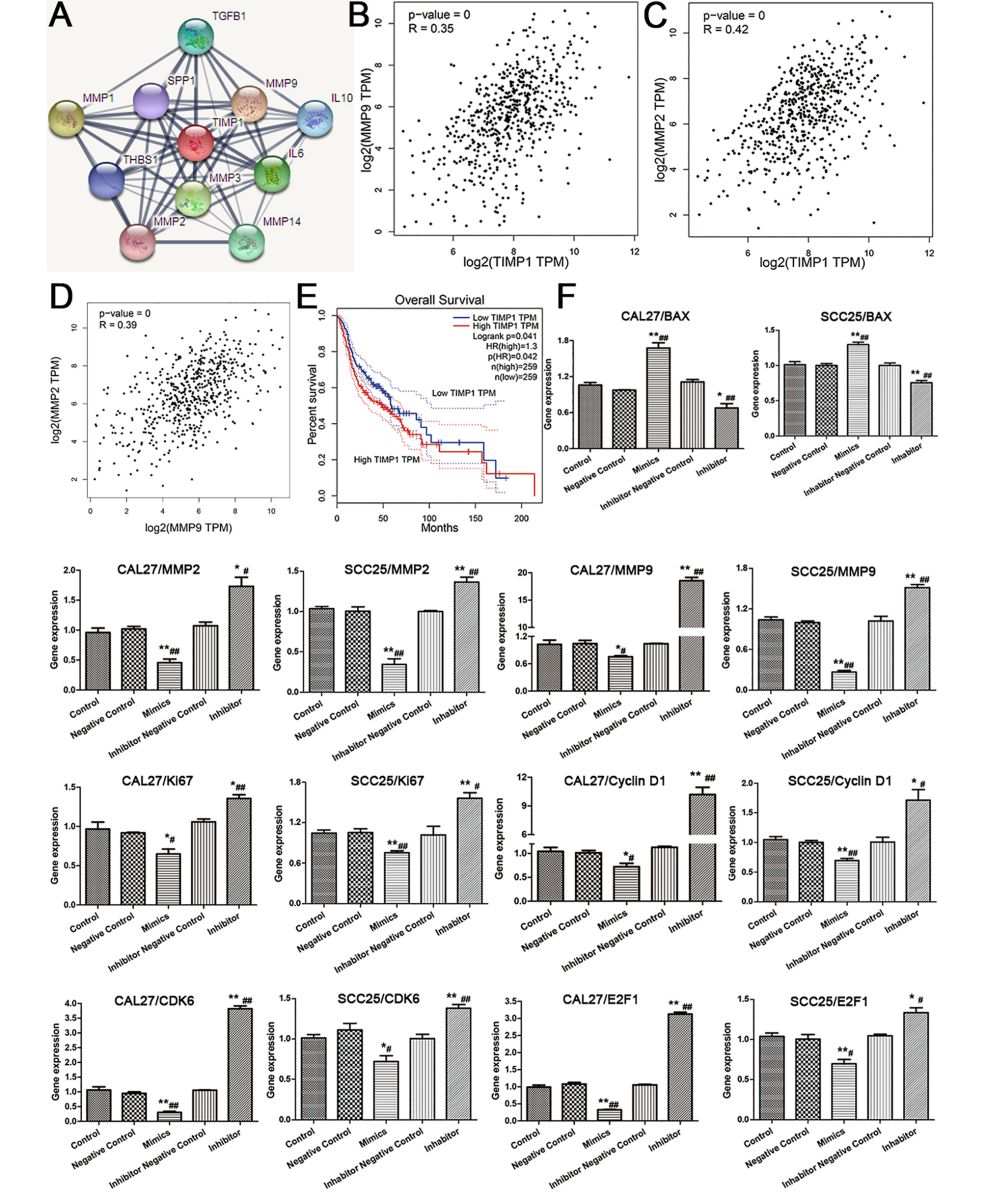
**(A)** The PPI network of TIMP1; **(B)** Correlation analysis of TIMP1 and MMP9; **(C)** Correlation analysis of TIMP1 and MMP2; **(D)** Correlation analysis of MMP9 and MMP2; **(E)** The survival curve of TIMP1; **(F)** The mRNA expression of downstream genes (n = 3)

PPI analysis indicated potential interactions between *TIMP1*, *MMP2*, and *MMP9* (Fig. 6A). Based on GEPIA analysis, *TIMP1* positively correlated with *MMP9* and *MMP2* (Fig. 6B, C), and *MMP9* positively correlated with *MMP2* (Fig. 6D). Additionally, patients with high *TIMP1* expression had low survival rates in head and neck squamous cell carcinoma (HNSCC) (Fig. 6E).

#### Differential expression of miR-181a-5p affected its downstream gene expression in OSCC cell lines

To further explore the regulatory mechanism of miR-181a-5p, we verified the expression levels of genes associated with cell cycle and proliferation (*KI67*, *CYCLIND1*, *CDK6*, and *E2F1*), migration and invasion (*TIMP1*, *MMP2*, and *MMP9*), and apoptosis (*BCL2* and *BAX*) in two transfected OSCC cell lines. In the miR-181a-5p mimic group, *BAX* was upregulated, while *TIMP1*, *MMP2*, *MMP9*, *BCL2*, *KI67*, *CYCLIND1*, *CDK6*, and *E2F1* were downregulated. In contrast, in the inhibitor group, the expression of these genes was opposite to that in the mimic group (Fig. 6F).

### miR-181a-5p suppresses tumor growth in vivo

#### Expression of miR-181a-5p in stable cell line

**Fig. 7:**
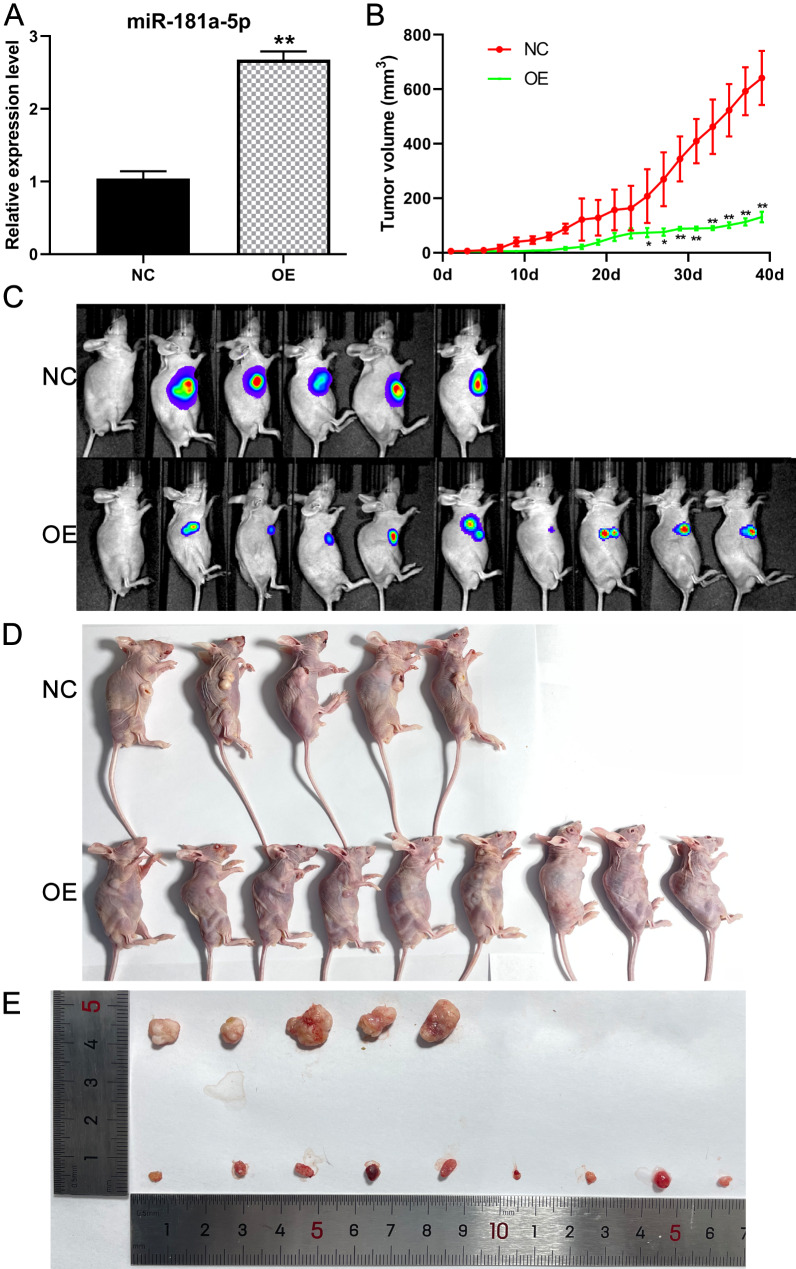
**(A)** The expression of miR-181a-5p in the stable cell line (n = 3); **(B)** Tumor growth curve of the nude mice xenograft model (NC: n = 5; OE: n = 9); **(C)** In vivo fluorescence imaging of the nude mice xenograft model; **(D)** Appearance picture of the nude mice xenograft model; **(E)** Isolated tumor observation of the xenograft model. NC, negative control group; OE, miR-181a-5p high-expression group. * P < 0.05 and ** P < 0.01

qPCR analysis indicated that the expression of miR-181a-5p was significantly upregulated in the OE group compared to that in the NC group (Fig. 7A).

#### miR-181a-5p inhibited cell growth in nude mice OSCC model

The tumor formation rates were 100% in the NC group (10/10) and 90% in the OE group (9/10). At the checkpoint, five nude mice were left in the NC group (all five mice had tumors), and 10 in the OE group (nine mice had tumors). The results indicated that tumor volumes were smaller in the OE group than in the NC group (Fig. 7B). Further, in vivo imaging demonstrated that the tumor volume and fluorescence values were significantly decreased in the OE group compared to that in the NC group (Fig. 7C). Moreover, the appearance and isolated tumor observation showed that the tumor volume in the OE group was much smaller than that in the NC group (Fig. 7D and E). The in vivo tumor growth results were consistent with the results of the cell experiment, which indicated that miR-181a-5p had a significant inhibitory effect on OSCC tumors.

**Fig. 8:**
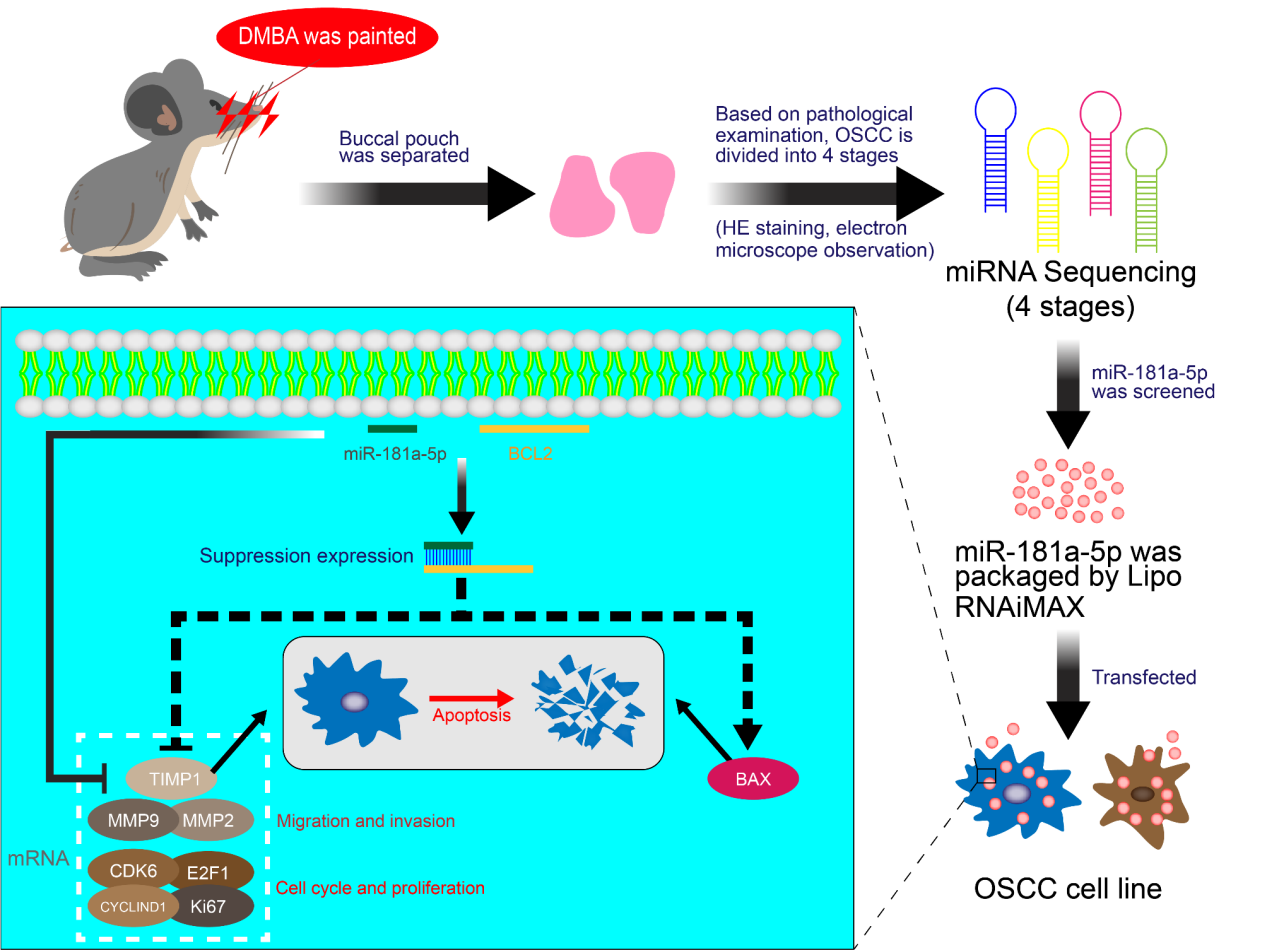
Schematic of the entire experiment

The schematic diagram of the entire experiment is shown in Fig. 8.

## Discussion

OSCC readily metastasizes and is aggressive, associated with a poor prognosis and a low survival rate. The clinical treatment and prognosis of OSCC are mainly based on pathological classification; however, this classification does not provide detailed information regarding therapeutic biology and related molecular mechanisms. Obtaining a clinical sample that meets experimental requirements is a major challenge in oral cancer research. Thus, a suitable animal model may help elucidate the molecular mechanisms underlying OSCC [30, 31]. Establishing an appropriate OSCC animal model is urgently required for further mechanistic research. The hamster buccal pouch (HBP) is covered by a thin layer of keratinized stratified squamous epithelium similar in thickness to the mouth mucosa and ventral surface of the tongue in humans [6]. The HBP model is one of the best-characterized and most useful cancer models for OSCC [32]. The earliest established OSCC animal model for HBP was reported by Salley in 1954 [33]. HBP has become a widely used animal model for oral carcinoma [34, 35]. Notably, the HBP model reflects many essential features of OSCC occurrence and development and is a useful and promising animal model for OSCC research [34].

The Chinese hamster, a subspecies of hamster, also called the stripped-back hamster (*C. griseus*), has a special buccal pouch tissue covered by a thin layer of keratinized stratified squamous epithelium similar in thickness to the mouth mucosa and the ventral surface of the tongue in humans. The Chinese hamster has strong vitality and a small body (approximately 9 cm long), which makes it easy to perform research operations. Given the above distinctive advantages, the Chinese hamster could be used as an animal model for the research of oral disease mechanisms [7]. To identify potential mechanisms involved in the occurrence and development of OSCC, we established a dynamic Chinese hamster buccal pouch OSCC animal model. According to the WHO guidelines for histological typing of cancer and precancer of the oral mucosa, four groups were established: normal, simple hyperplasia, abnormal hyperplasia, and squamous cell carcinoma. Thereafter, we built miRNA libraries and utilized high-throughput sequencing and bioinformatics methods to examine miRNAs. We found that miR-181a-5p is significantly downregulated in human OSCC cell lines and tissues. Previous studies have also revealed that the expression level of miR-181a is reduced in OSCC [23, 36]. Thus, miR-181a-5p was selected for in vitro functional studies. The above findings support the role of miR-181a-5p in OSCC and demonstrate that the Chinese hamster buccal pouch OSCC model is indeed a useful tool for research on the cell biology, pathology, and therapeutics of OSCC.

Accumulating evidence suggests that changes in gene expression occur during the transformation of normal cells to malignant cells. In our animal model, sequencing and qPCR results showed that the expression of miR-181a-5p, a member of the miR-181a family, gradually declined in the simple hyperplasia, abnormal hyperplasia, and squamous cell carcinoma stages and reached a minimum level at the squamous cell carcinoma stage. Moreover, miR-181a-5p was downregulated in human OSCC cell lines and human OSCC specimens. Screening and validation of animal models, patient tissues, and cell lines showed that the decrease in miR-181a-5p expression may be related to changes in cell function. To better understand the role of miR-181a-5p, we investigated the biological mechanisms through which miR-181a-5p is involved in the regulation of OSCC cell lines. Functional experiments showed that miR-181a-5p could inhibit cell proliferation, colony formation, migration, invasion, and cell cycle and promote apoptosis. To further validate our results, we performed functional experiments using another OSCC cell line (SCC-25). Interestingly, the results showed that consistent with CAL-27, miR-181a-5p also has an obvious tumor suppressor role in the SCC-25 cell line.

To better explore the role of miR-181a-5p in suppressing tumors in vivo, we established a stable and highly expressing miR-181a-5p cell line and constructed a tumor xenograft animal model. The results demonstrated that miR-181a-5p significantly inhibited tumor growth in vivo. These findings indicate that miR-181a-5p could act as a tumor suppressor, which is consistent with the results of our in vitro animal model and cell line verification as well as previous studies [13, 37, 38]. Similarly, a study on nasopharyngeal carcinoma (NPC) showed that miR-181a expression was reduced in NPC. An increased miR-181a expression can retard the malignant development of NPC both in vivo and in vitro [39]. In addition, miR-181a negatively regulates NF-kB signaling and inhibits activated B-cell-like diffuse large B-cell lymphoma [19]; miR-181a-5p can also induce cellular senescence and shorten cellular lifespan [20, 21]. These results reflect the potential tumor suppressor function of miR-181a-5p, which is consistent with our results.

The orthotopic model can more accurately simulate the occurrence and development of oral cancer in humans; completely replicate the pathology, microenvironment, or steps of premalignancy and carcinogenesis in human oral cancer; and promote clinical applications [40]. Based on this, we first established a DMBA-induced orthotopic oral tumor model using the Chinese hamster and screened and validated the function of the candidate gene miR-181a-5p in vitro. This was followed by the establishment of a nude mouse subcutaneous xenograft tumor model to verify the in vivo function of miR-181a-5p. The location of tumor growth in subcutaneously transplanted tumor animal models is superficial, easy to operate on, and can be directly observed, facilitating their measurement and monitoring. In the present study, we considered that nude mice, which are small in size and have limited oral space, are extremely prone to additional damage to the oral cavity when undergoing orthotopic transplantation and cannot be directly observed to detect tumor growth. Currently, subcutaneous xenograft models are widely used to study the pathogenesis of oral cancer and in vivo functional studies of key genes [41–46]. Therefore, in this study, we considered the advantages of the combination of the two animal models, we first employed an orthotopic model to screen candidate miRNAs and their related genes and pathways. Subsequently, a subcutaneous xenograft model was used to validate the in vivo function of miR-181a-5p. In the future, we plan to establish an orthotopic model to further investigate the in vivo tumor suppressor mechanisms of miR-181a-5p.

We screened and validated *BCL2* as the downstream target of miR-181a-5p. Ghoshal-Gupta et al. [47] and Srilatha et al. [48] revealed an interaction between *BCL2* and *TIMP1*. Srilatha et al. [48] verified that *BCL2* can interact with *TIMP1* using a co-immunoprecipitation (co-IP) experiment. *TIMP1* is highly expressed in multiple tumors and interacts with members of the *MMP* family [49–51]. According to our qPCR and western blot results, miR-181a-5p also affected the expression of *BCL2* and *TIMP1*. Additionally, high *TIMP1* levels were associated with poor prognosis in patients with HNSCC. Ruokolainen et al. showed that tissue overexpression or elevated *TIMP1* in the serum is associated with poor prognosis in many tumor types, including HNSCC [52]. In the present study, the STRING database was used to analyze the PPI of TIMP1, and the results indicated that TIMP1 potentially interacted with MMP2 and MMP9. GEPIA database analyses showed that there were potential correlations between TIMP1 and MMP2, TIMP1 and MMP9, and MMP9 and MMP2. Furthermore, in a study on the progress and invasion of oral cancer, the author also used a co-IP experiment to prove the interaction between TIMP1 and MMP9 [49]. In conclusion, based on the database and literature verification, it was confirmed that there are potential interactions between BCL2 and TIMP1, TIMP1 and MMP9, and MMP9 and MMP2. Therefore, we speculated that miR-181a-5p may function by interacting with its target and downstream genes.

*MMP2* and *MMP9* are related to invasion and migration [53, 54]. Abdollahi et al. demonstrated that the mRNA level of *TIMP1* was considerably higher in patients with axillary lymph nodes than in patients without metastasis [55]. In a study by Kamdeo et al., the upregulation and activation of *MMP9* were observed at various stages of oral tumors, while positive correlations were observed between *MMP9* and *MMP2* activity, *MMP9* and *TIMP1* expression, and *TIMP1*-*MMP9* interaction [49, 56]. Yu et al. also demonstrated that ERK1/2 and Akt signaling pathways mediate the amphiregulin-induced upregulation of *MMP9* and *TIMP1*, subsequently promoting HTR-8/SVneo cell invasion [50]. In addition to its interaction with *MMPs*, *TIMP1* has potent growth-promoting activity in several types of cells [57–59]. *TIMP1* is also recognized as a cancer-promoting factor due to its anti-apoptotic effects [48, 60]. *TIMP1* promotes cell growth and survival [61], mitogenic activity [62], and angiogenesis [63]. Based on the above studies and our results, we speculate that the differentially expressed miR-181a-5p regulates the expression of *BCL2* and subsequently affects the expression of *TIMP1*, *MMP2*, and *MMP9* to further regulate migration and invasion in vitro. Co-IP experiments were not performed in this study to verify the above-mentioned interaction relationships. In follow-up studies, we will further investigate the relationship between these key genes in oral cancer.

Gong et al. reported that miR-181a targets *BCL2* in human malignant glioma U87MG cells [64]. In breast cancer, miR-181a-5p can significantly downregulate *BCL2*, leading to breast cancer cell apoptosis [65]. Nalluri et al. demonstrated a co-dependent relationship between *BCL2* and *TIMP1*. Interestingly, their research on apoptosis proved that *TIMP1* leads to the phosphorylation of the pro-apoptotic protein BAD, ultimately decreasing *BAX* levels and increasing mitochondrial permeability [48]. Based on the findings of these studies and our results, miR-181a-5p may regulate cell apoptosis by directly or indirectly acting on *BCL2* or *TIMP1*.

Cell cycle disorders can lead to excessive cell proliferation, an important cause of human cancer [66]. Based on further investigations, miR-181a-5p may affect the cell cycle and proliferation of OSCC cell lines by regulating the expression of cycle/proliferation-related genes, such as *KI67*, *CYCLIND1*, *CDK6*, and *E2F1*. *CYCLIND1* regulatory subunits and CDK binding form functional complexes that significantly affect cell proliferation [67]. The *E2F1* transcription factor plays an important role in cell proliferation, differentiation, and apoptosis [68, 69]. Previously, *pRB* was demonstrated to be phosphorylated by the *CDK4/6*-*CYCLIND* complex, leading to the activation of *E2F* and, ultimately, the regulation of cell cycle progression [70]. These findings indicate that miR-181a-5p may affect the expression of *CYCLIND1*, further affecting the formation of *CYCLIND1*/*CDK6* functional complexes. As a result, the activation of the transcription factor *E2F1* and its downstream genes is inhibited, ultimately leading to the regulation of OSCC cell proliferation and cell cycle.

The Chinese hamster oral cancer model can grow tumors spontaneously in the oral mucosa, enabling a better simulation of the occurrence of human oral tumors than other models. In the present study, animal models of four stages of oral cancer were constructed, and we found that miR-181a-5p may act as a potential tumor suppressor in oral cancer. Therefore, miR-181a-5p was screened for functional validation. Subsequently, we validated the expression of miR-181a-5p in OSCC tissues and multiple cell lines. Furthermore, a tumor xenograft animal model and suitable oral cancer cell line were used to investigate the expression, function, and potential molecular mechanisms of miR-181a-5p in vivo and in vitro. According to the results, miR-181a-5p may inhibit tumor growth in vivo; inhibit cell proliferation, colony formation, migration, invasion, and the cell cycle; and promote the apoptosis of OSCC cells in vitro by affecting the corresponding downstream genes. In addition, a tumor xenograft animal model revealed that miR-181a-5p suppresses tumor growth in vivo.

## Conclusion

In summary, the results indicate that the Chinese hamster has potential as a novel oral cancer animal model, and these findings elucidate the possible regulatory mechanism of miR-181a-5p in oral cancer. These results, together with the novel Chinese hamster buccal pouch animal model, are expected to contribute to further research on the mechanisms of occurrence and development of oral cancer. In future studies, we will further explore and verify the role of miR-181a-5p in the occurrence, development, treatment, and prognosis of OSCC by comprehensively combining clinical samples with diagnostic and therapeutic data.

## Electronic supplementary material

Below is the link to the electronic supplementary material.

Supplementary Material 1

Supplementary Material 2

Supplementary Material 3

Supplementary Material 4

Supplementary Material 5

Supplementary Material 6

## Data Availability

The datasets used and/or analyzed during the current study are available from the corresponding author upon reasonable request. The datasets generated and/or analysed during the current study are available in the Gene Expression Omnibus (GEO) repository (GSE222429).
