# Supplementary material for: Screening and identification of miR-181a-5p in oral squamous cell carcinoma and functional verification in vivo and in vitro
Source: BMC Cancer. 2023 Feb 17;23:162. doi: 10.1186/s12885-023-10600-3 (PMC9936757; doi:10.1186/s12885-023-10600-3)
Supplement: Supplementary file 1 — Supplementary Material 1 [file 12885_2023_10600_MOESM1_ESM.docx]

Supplementary table 1. Information of the primer sequence

| Name | Sequence（5'-3'） |
| --- | --- |
| miR-181a-5p | CATTCAACGCTGTCGGTGAGTA |
| miR-450b-5p | TTTTGCAGTATGTTCCTGAATAAAA |
| miR-142-5p | GCCCATAAAGTAGAAAGCACTACAA |
| miR-33 | GTGCATTGTAGTTGCATTGCAA |
| miR-340-5p | CCTTATAAAGCAATGAGACTGATTAAA |
| miR-20a | CTAAAGTGCTTATAGTGCAGGTAGAAA |
| miR-210-5p | CTGCCCACCGCACACTGA |
| miR-23a-5p | GTTCCTGGGGATGGGATTTA |
| miR-1260 | CACCGCTGCCACCAAAA |
| miR-191-5p | GGAATCCCAAAAGCAGCTGA |
| miR-15b-5p | TAGCAGCACATCATGGTTTACAAA |
| miR-34c-5p | AGGCAGTGTAGTTAGCTGATTGC |
| miR-504 | AGACCCTGGTCTGCACCTCTA |
| miR-32-5p | CCTATTGCACATTACTAAGTTGCAA |
| U6 | CGCTTCGGCAGCACATATAC |
| MMP9 | F: CTTTGGACACGCACGAC |
|  | R: CCACCTGGTTCAACTCACT |
| MMP2 | F: CAAGGACCGGTTCATTTGGC |
|  | R: GGCCTCGTATACCGCATCAA |
| KI67 | F: GTGCGAAGGTTCTCATGC |
|  | R: CTTGACACTCCGCGTTAC |
| BCL2 | F: ACTGGCTCTGTCTGAGTAAG |
|  | R: CCTGATGCTCTGGGTAAC |
| BAX | F: TGGCAGCTGACATGTTTTCTGAC |
|  | R: TCACCCAACCACCCTGGTCTT |
| TIMP1 | F: CATTGCTGGAAAACTGCAGGA |
|  | R: TCCACAAGCAATGAGTGCCA |
| CYCLIN D1 | F: ATGTTCGAGGCGCGCCTGGTC |
|  | R: CTAAGATCCTTCTTCATCCTC |
| CDK6 | F: GTGACCAGCAGCGGACAAATAA |
|  | R: AGCAAGACTTCGGGTGCTCTGTA |
| E2F1 | F: AGCTGGACCACCTGATGAATATCTG |
|  | R: TTGATCACCATAACCATCTGCTCTG |
| GAPDH | F: GCACCGTCAAGGCTGAGAAC |
|  | R: TGGTGAAGACGCCAGTGGA |
