# Supplementary material for: Screening and identification of miR-181a-5p in oral squamous cell carcinoma and functional verification in vivo and in vitro
Source: BMC Cancer. 2023 Feb 17;23:162. doi: 10.1186/s12885-023-10600-3 (PMC9936757; doi:10.1186/s12885-023-10600-3)
Supplement: Supplementary file 2 — Supplementary Material 2 [file 12885_2023_10600_MOESM2_ESM.docx]

Supplementary table 2. Specific clinical features of patient samples

| Group | Gender | Age | Differentiation | Stages | Lymph node metastasis |
| --- | --- | --- | --- | --- | --- |
| Sample 1 | male | 61 | moderately | Ш | no |
| Sample 2 | male | 67 | well | Ⅳ | no |
| Sample 3 | male | 59 | moderately | Ⅱ | no |
| Sample 4 | male | 77 | moderately | Ш | yes |
| Sample 5 | male | 63 | well | Ⅳ | no |
| Counterpart 1 | male | 61 | — | — | — |
| Counterpart 2 | male | 67 | — | — | — |
| Counterpart 3 | male | 59 | — | — | — |
