# Supplementary material for: Screening and identification of miR-181a-5p in oral squamous cell carcinoma and functional verification in vivo and in vitro
Source: BMC Cancer. 2023 Feb 17;23:162. doi: 10.1186/s12885-023-10600-3 (PMC9936757; doi:10.1186/s12885-023-10600-3)
Supplement: Supplementary file 4 — Supplementary Material 4 [file 12885_2023_10600_MOESM4_ESM.docx]

Supplementary table 4. The information of DEmiRNA between the abnormal hyperplasia and simple hyperplasia groups

| miRNA | countMean | log2FoldChange | pvalue | padj | threshold |
| --- | --- | --- | --- | --- | --- |
| miR-21-5p | 361713.4 | 2.964886 | 2.91E-22 | 4.04E-20 | Up |

Supplementary table 5. The information of DEmiRNAs between the squamous cell carcinoma and simple hyperplasia groups

| miRNA | countMean | log2FoldChange | pvalue | padj | threshold |
| --- | --- | --- | --- | --- | --- |
| miR-27a-5p | 551.3426 | 3.994425 | 1.21E-34 | 1.68E-32 | Up |
| miR-21-5p | 361713.4 | 3.576522 | 1.19E-31 | 8.29E-30 | Up |
| miR-31-5p | 1013.769 | 4.246738 | 7.96E-14 | 3.69E-12 | Up |
| miR-146b-5p | 3710.38 | 2.021523 | 5.89E-09 | 2.05E-07 | Up |
| miR-100-5p | 24421.04 | -1.16308 | 2.07E-07 | 5.74E-06 | Down |
| miR-1260 | 1725.711 | 2.074334 | 1.03E-05 | 0.000238 | Up |
| let-7d-5p | 5517.588 | -1.19198 | 5.74E-05 | 0.001088 | Down |
| miR-92a-5p | 177.852 | 1.584291 | 6.81E-05 | 0.001088 | Up |
| miR-193b-5p | 30.6102 | -2.45206 | 7.05E-05 | 0.001088 | Down |
| miR-99a-5p | 209180.9 | -1.11486 | 9.23E-05 | 0.001283 | Down |
| miR-384-5p | 40.83505 | -2.03619 | 0.000121 | 0.001524 | Down |
| miR-331-5p | 40.98803 | 2.424797 | 0.000138 | 0.001603 | Up |
| miR-455-5p | 2748.629 | 1.870989 | 0.000525 | 0.0056 | Up |
| miR-378-5p | 1083.078 | -1.2497 | 0.000601 | 0.0056 | Down |
| miR-450a | 238.7583 | 1.096057 | 0.000606 | 0.0056 | Up |
| miR-181a-5p | 12220.33 | -1.01608 | 0.000886 | 0.006845 | Down |
| miR-365-5p | 111.6065 | -1.53729 | 0.001084 | 0.007532 | Down |
| miR-144 | 126.7103 | 1.975118 | 0.001443 | 0.009554 | Up |
| miR-181d-5p | 377.0615 | 1.14455 | 0.001563 | 0.009874 | Up |
| miR-26a-2 | 260555.2 | -1.18903 | 0.00275 | 0.016621 | Down |
| miR-132-5p | 113.351 | 1.106588 | 0.002937 | 0.016864 | Up |
| miR-190a | 63.1113 | -1.10873 | 0.003033 | 0.016864 | Down |
| miR-155 | 1390.109 | 1.018973 | 0.003369 | 0.018012 | Up |
| miR-26b-5p | 27498.4 | -1.12746 | 0.003742 | 0.018576 | Down |
| miR-34b-5p | 1060.646 | -1.07096 | 0.003731 | 0.018576 | Down |
| miR-450b-5p | 76.45365 | 1.321717 | 0.004264 | 0.019755 | Up |
| miR-23b-5p | 52.90764 | 1.134736 | 0.005892 | 0.02642 | Up |
| miR-130b-5p | 6.26394 | 2.107371 | 0.007372 | 0.032021 | Up |
| miR-212 | 6.84552 | 1.885983 | 0.008872 | 0.037371 | Up |
| miR-499-5p | 124.8437 | -1.08227 | 0.009156 | 0.037431 | Down |
| miR-138 | 12.61273 | -1.90953 | 0.010003 | 0.037579 | Down |
| miR-152-5p | 148.0546 | 1.016134 | 0.009547 | 0.037579 | Up |
| miR-129 | 25.99481 | 1.440109 | 0.010312 | 0.03772 | Up |
| miR-134 | 39.6615 | 1.375287 | 0.013399 | 0.045426 | Up |
| miR-29b-5p | 7.779532 | 2.570603 | 0.014862 | 0.046951 | Up |

Supplementary table 6. The information of DEmiRNAs between the squamous cell carcinoma and abnormal hyperplasia groups

| miRNA | countMean | log2FoldChange | pvalue | padj | threshold |
| --- | --- | --- | --- | --- | --- |
| miR-21-5p | 361713.4 | 2.964886 | 2.91E-22 | 4.04E-20 | Up |
| miR-27a-5p | 551.3426 | 2.729785 | 2.49E-17 | 1.73E-15 | Up |
| miR-31-5p | 1013.769 | 4.04834 | 1.17E-12 | 5.44E-11 | Up |
| miR-100-5p | 24421.04 | -1.16013 | 2.23E-07 | 7.73E-06 | Down |
| miR-99a-5p | 209180.9 | -1.22203 | 1.82E-05 | 0.000506 | Down |
| miR-499-5p | 124.8437 | -1.65966 | 6.56E-05 | 0.001521 | Down |
| miR-181d-5p | 377.0615 | 1.364796 | 0.000179 | 0.003549 | Up |
| miR-384-5p | 40.83505 | -1.92005 | 0.000334 | 0.005805 | Down |
| miR-92a-5p | 177.852 | 1.355329 | 0.000728 | 0.009363 | Up |
| miR-146b-5p | 3710.38 | 1.171858 | 0.000741 | 0.009363 | Up |
| miR-322-5p | 192.2224 | 1.403327 | 0.000668 | 0.009363 | Up |
| miR-450a | 238.7583 | 1.053502 | 0.001062 | 0.012304 | Up |
| miR-10a-5p | 873.7061 | -1.88245 | 0.00131 | 0.013569 | Down |
| miR-455-5p | 2748.629 | 1.727837 | 0.001367 | 0.013569 | Up |
| miR-193b-5p | 30.6102 | -2.00292 | 0.001684 | 0.015602 | Down |
| miR-450b-5p | 76.45365 | 1.394815 | 0.002897 | 0.023686 | Up |
| miR-365-5p | 111.6065 | -1.31593 | 0.005665 | 0.037497 | Down |
| miR-130b-5p | 6.26394 | 2.281884 | 0.007711 | 0.045252 | Up |
