# Supplementary material for: Screening and identification of miR-181a-5p in oral squamous cell carcinoma and functional verification in vivo and in vitro
Source: BMC Cancer. 2023 Feb 17;23:162. doi: 10.1186/s12885-023-10600-3 (PMC9936757; doi:10.1186/s12885-023-10600-3)

1-CAL27-actin：C, NC, M, IN, I


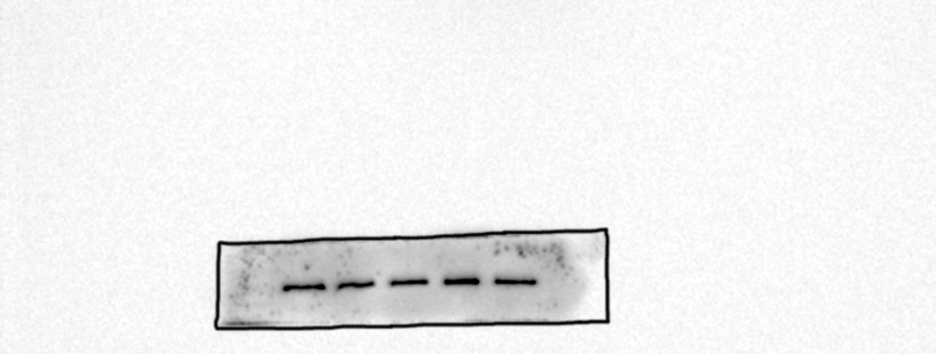


1-CAL27-BCL2：C, NC, M, IN, I


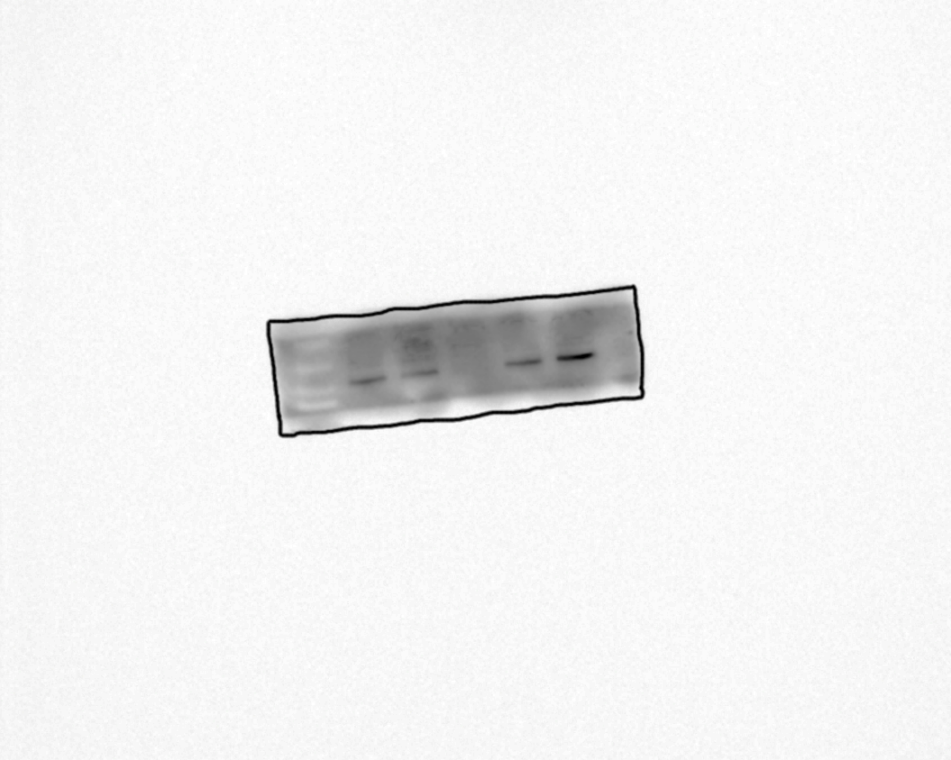


1-CAL27-TIMP1：C, NC, M, IN, I


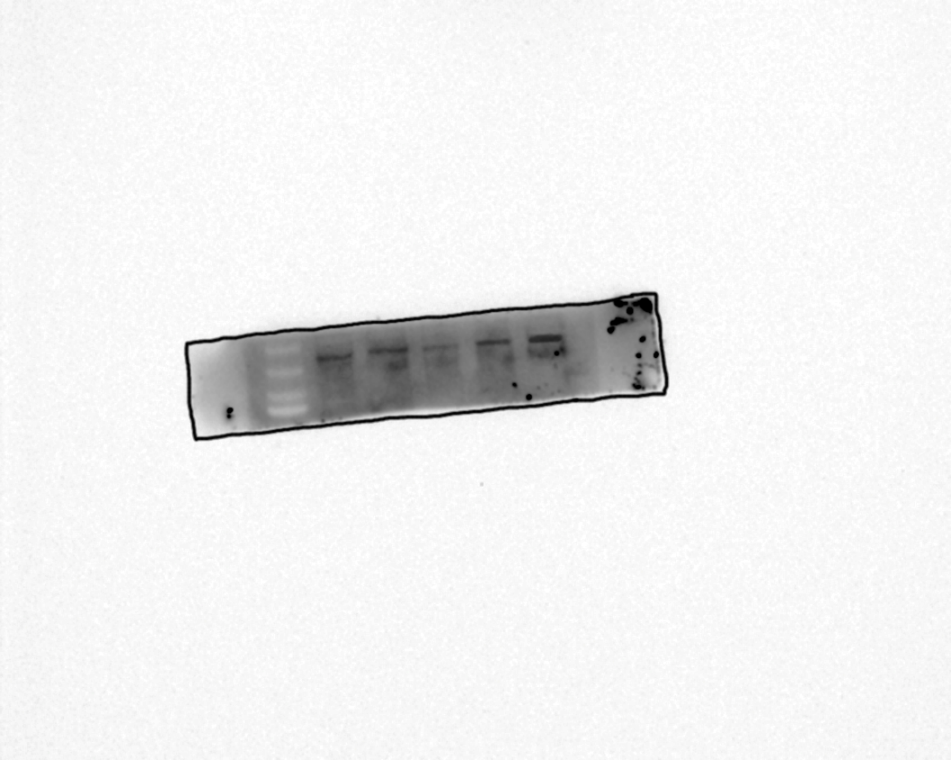


2-CAL27-actin


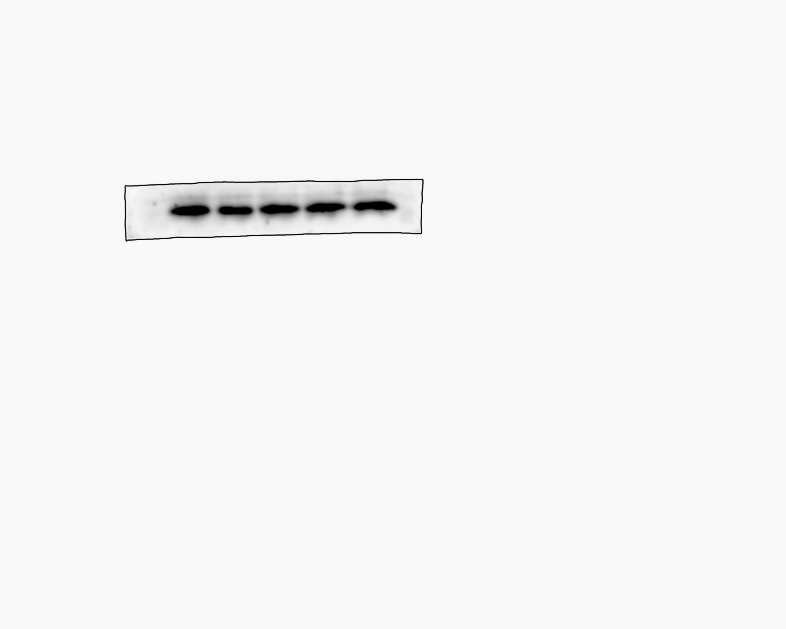


2-CAL27-BCL2


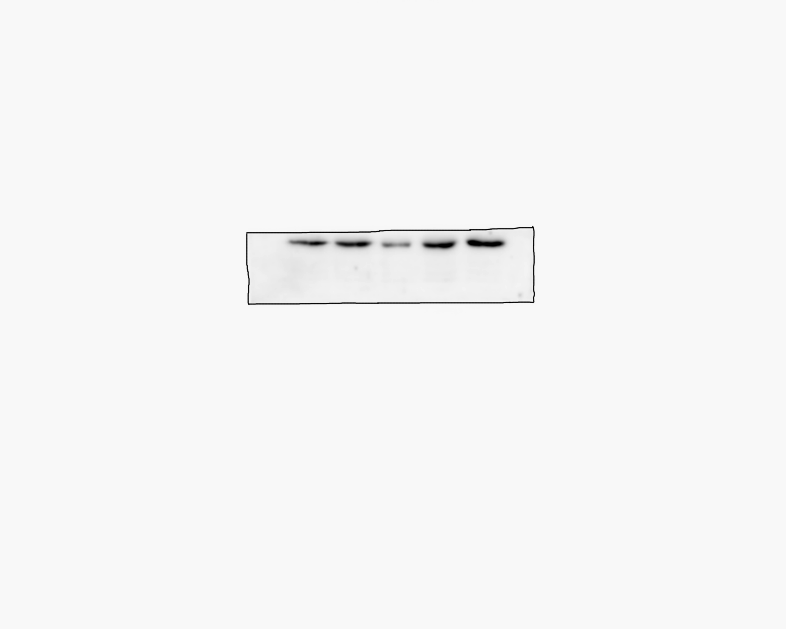


2-CAL27-TIMP1


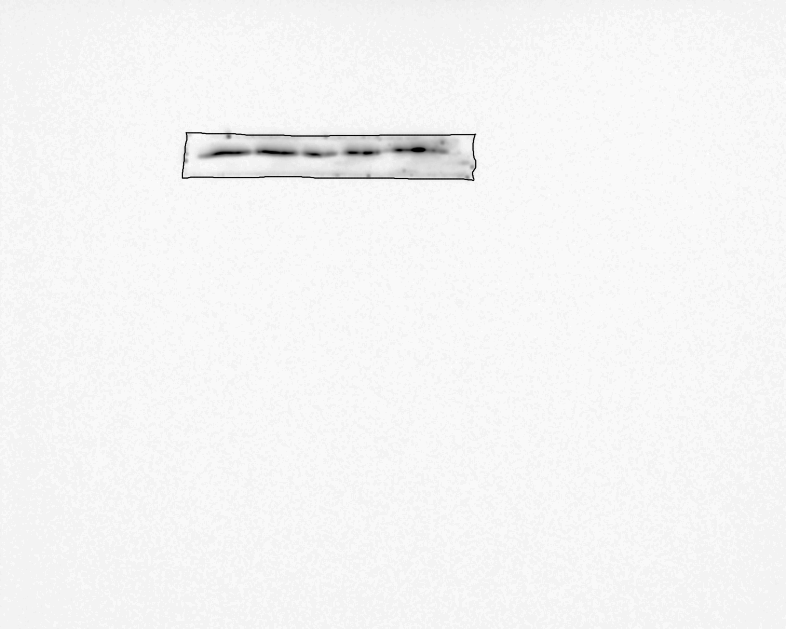


3-CAL27-actin


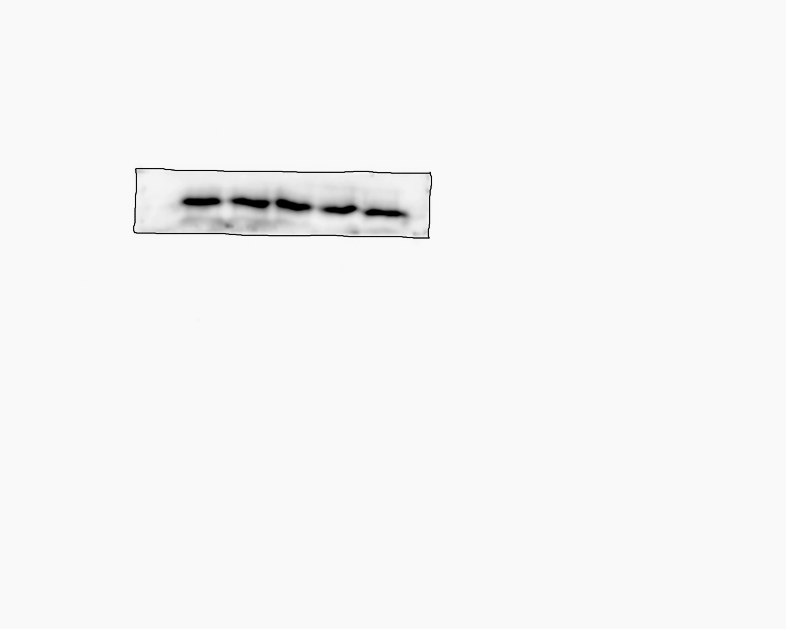


3-CAL27-BCL2


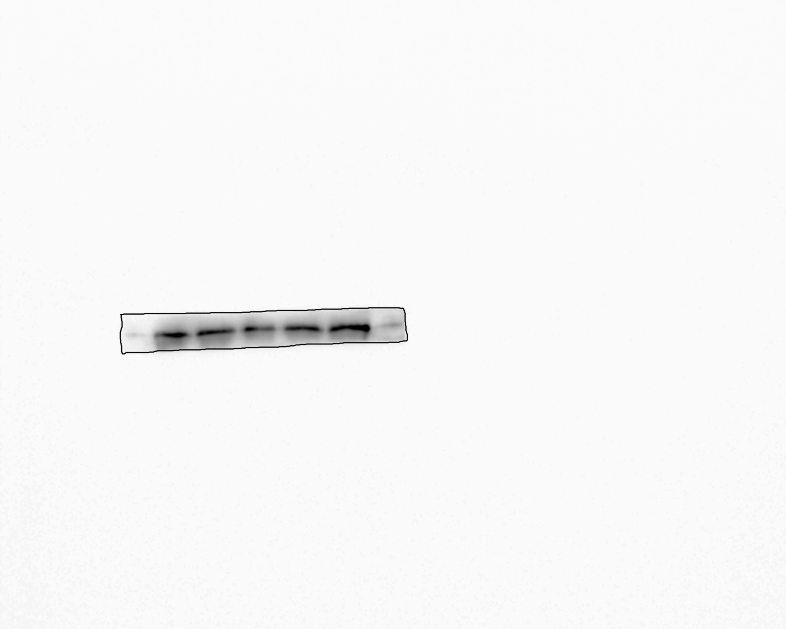


3-CAL27-TIMP1


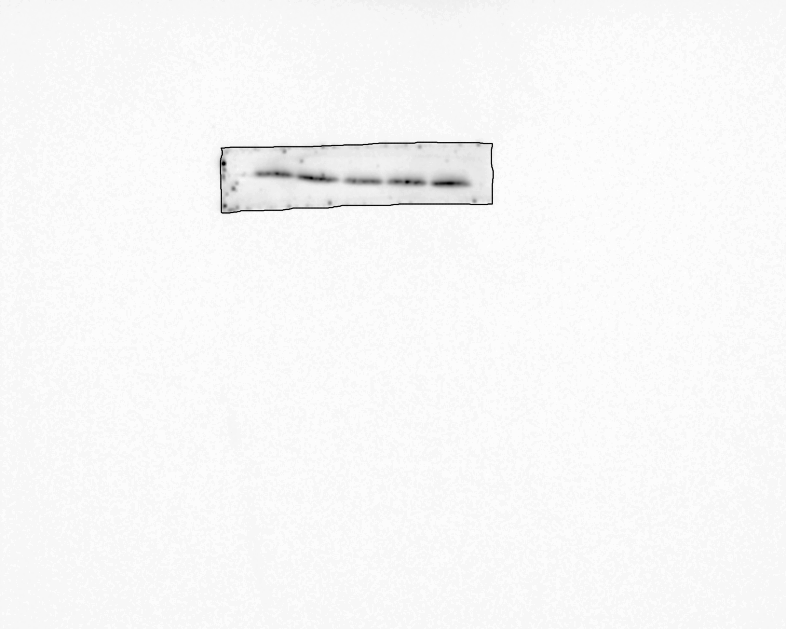


1-SCC25-actin


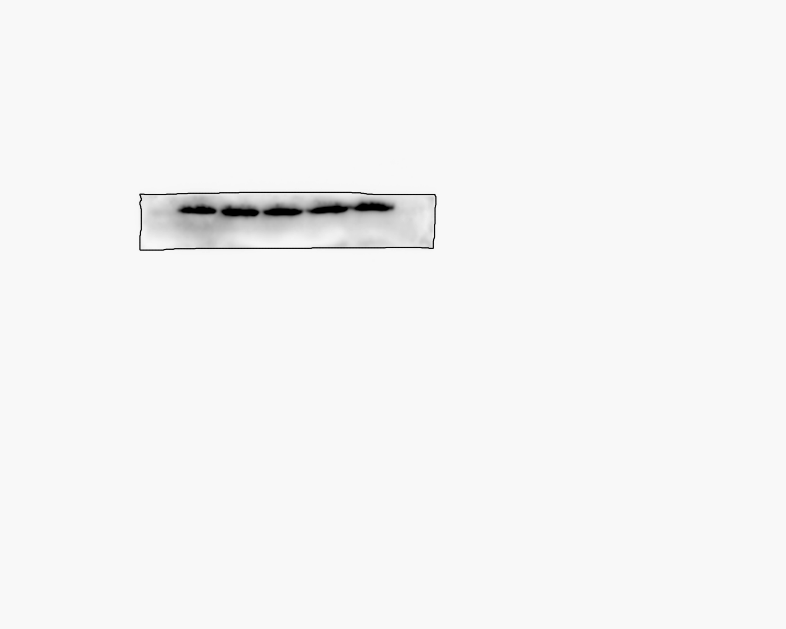


1-SCC25-BCL2


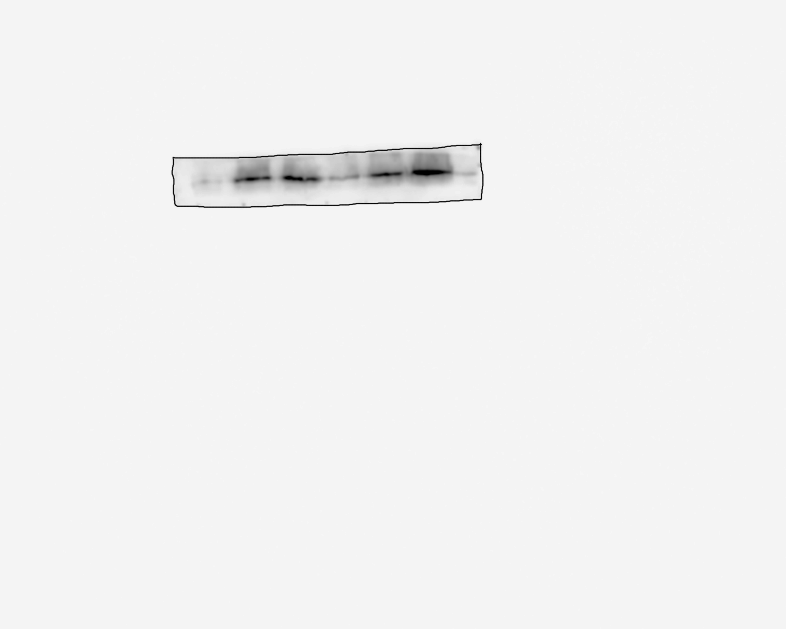


1-SCC25-TIMP1


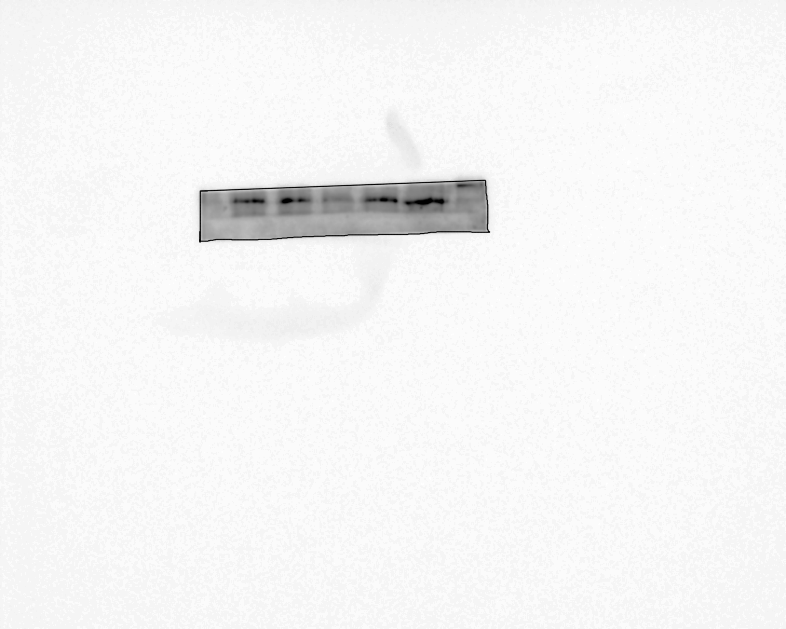


2-SCC25-actin


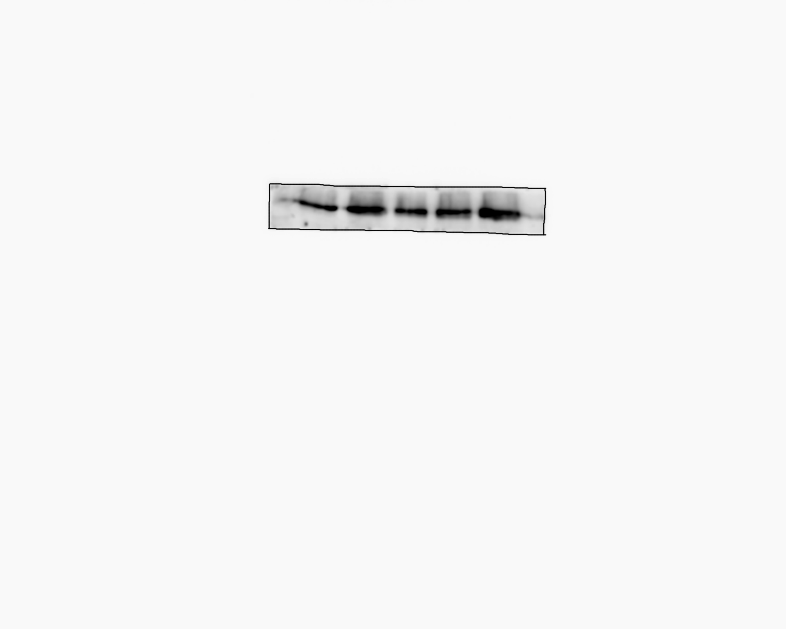


2-SCC25-BCL2


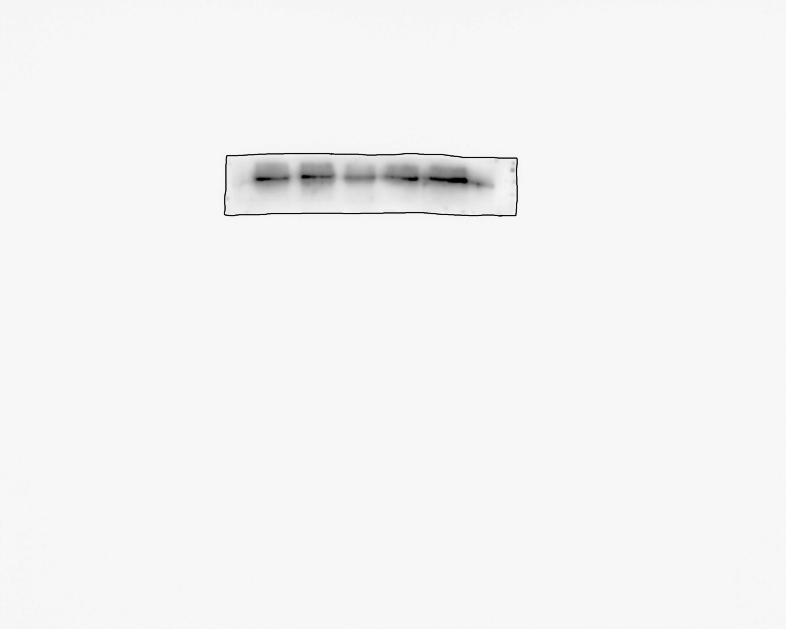


2-SCC25-TIMP1


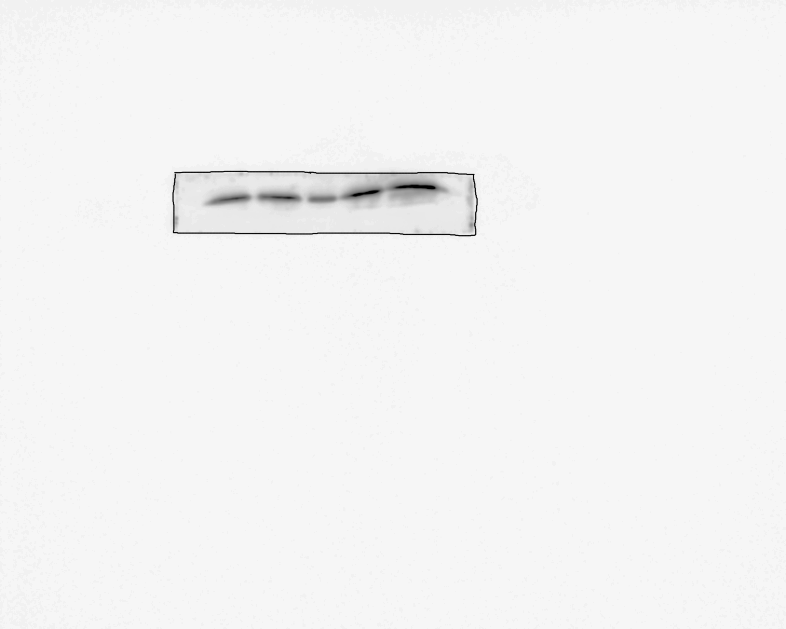


3-SCC25-actin


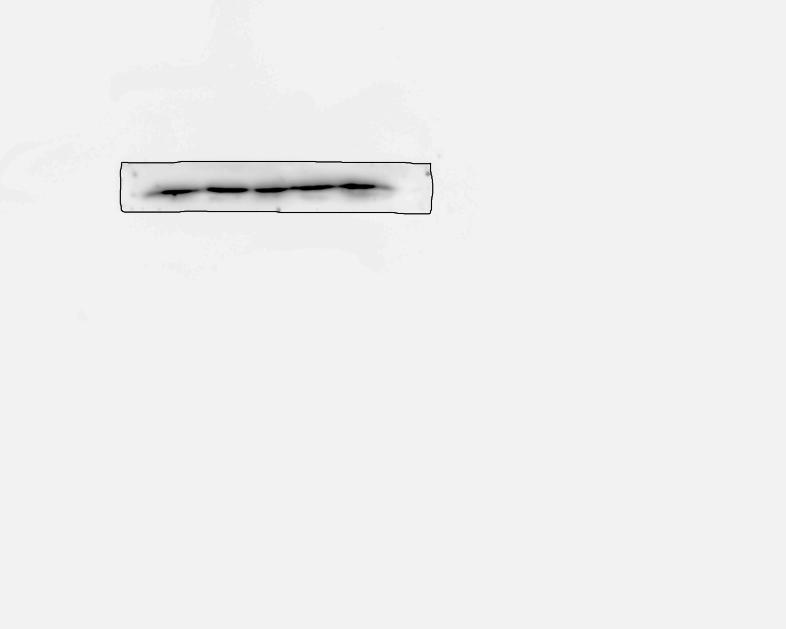


3-SCC25-BCL2


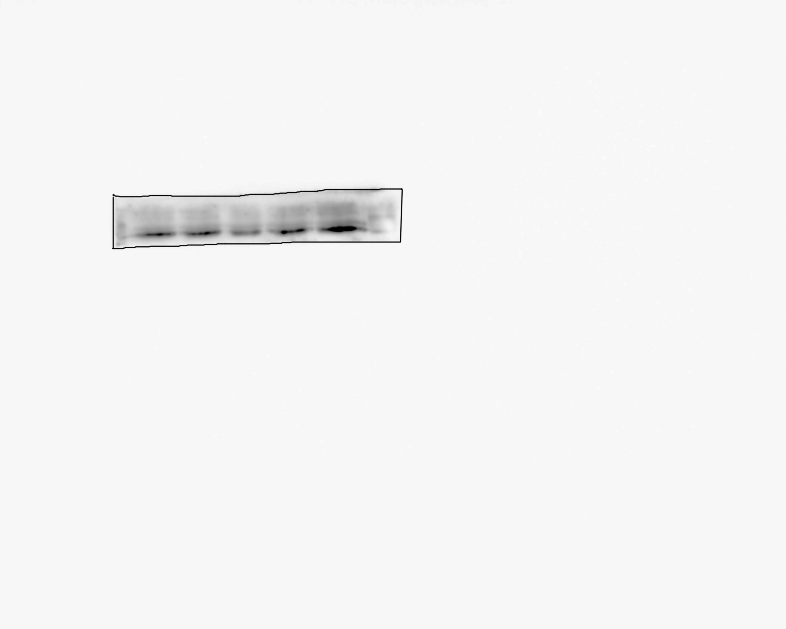


3-SCC25-TIMP1


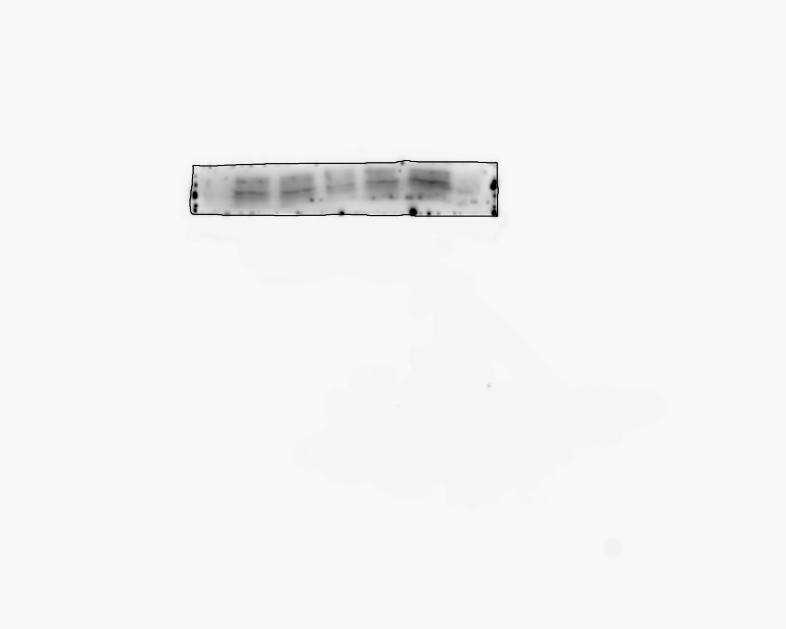

Supplement: Supplementary file 6 — Supplementary Material 6 [file 12885_2023_10600_MOESM6_ESM.docx]
